# Mapping Background Variables With Sequential Patterns in Problem-Solving Environments: An Investigation of United States Adults’ Employment Status in PIAAC

**DOI:** 10.3389/fpsyg.2019.00646

**Published:** 2019-03-27

**Authors:** Dandan Liao, Qiwei He, Hong Jiao

**Affiliations:** ^1^Department of Human Development and Quantitative Methodology, University of Maryland, College Park, MD, United States; ^2^Educational Testing Service, Princeton, NJ, United States

**Keywords:** process data, problem solving, sequential pattern, background variables, large-scale assessment PIAAC

## Abstract

Adult assessments have evolved to keep pace with the changing nature of adult literacy and learning demands. As the importance of information and communication technologies (ICT) continues to grow, measures of ICT literacy skills, digital reading, and problem-solving in technology-rich environments (PSTRE) are increasingly important topics for exploration through computer-based assessment (CBA). This study used process data collected in log files and survey data from the Programme for the International Assessment of Adult Competencies (PIAAC), with a focus on the United States sample, to (a) identify employment-related background variables that significantly related to PSTRE skills and problem-solving behaviors, and (b) extract robust sequences of actions by subgroups categorized by significant variables. We conducted this study in two phases. First, we used regression analyses to select background variables that significantly predict the general PSTRE, literacy, and numeracy skills, as well as the response time and correctness in the example item. Second, we identified typical action sequences by different subgroups using the chi-square feature selection model to explore these sequences and differentiate the subgroups. Based on the malleable factors associated with problem-solving skills, the goal of this study is to provide information for improving competences in adult education for targeted groups.

## Introduction

Adult assessments have evolved to keep pace with the changing nature of adult literacy and learning demands. As the importance of information and communication technologies (ICT) continues to grow, measures of ICT skills are increasingly important topics for exploration through computer-based assessment (CBA). The Programme for the International Assessment of Adult Competencies (PIAAC) is the first international household survey of adult skills predominantly collected using ICT skills. Conducted in 40 countries, this international survey measures key cognitive and workplace skills including literacy, numeracy, and problem-solving in technology-rich environments (PSTRE). These skills are not only critical to individual prosperity but are also key drivers of economic growth and societal advancement (Organisation for Economic Co-operation and Development [OECD], 2013b, p. 3).

Specifically, PSTRE assessment focuses on the ability of “using digital technology, communication tools and networks to acquire and evaluate information, communicate with others and perform practical tasks” (Organisation for Economic Co-operation and Development [OECD], 2012). As digital technology has become an indispensable part of human lives, there is an increasing need for measuring the ability to solve problems in conjunction with basic computer literacy skills. PSTRE assessment renders it possible to measure how well adults process, analyze, and address problems for specific goals in a computer-based environment.

According to a recent report published by the National Center for Education Statistics (Rampey et al., 2016), United States respondents on average scored lower than respondents from other countries in the PSTRE domain (Organisation for Economic Co-operation and Development [OECD], 2013b, p. 11). In particular, the United States sample had the largest proportion of respondents scoring below Level 1, which is the minimum proficiency level required to complete simple problem-solving tasks in daily life (Organisation for Economic Co-operation and Development [OECD], 2013b, p. 21).

Some facts about specific subgroups of United States respondents are also concerning. Scores for millennials (adults born after 1980 and between ages 16–34) in the United States were among the lowest of all participating countries even though over half of them spent 35 hours per week on digital media (Organisation for Economic Co-operation and Development [OECD], 2013b, p. 21; Goodman et al., 2015). It was found that 41% of respondents with less than high school education chose to take the paper version of PIAAC, compared to 17% for high school graduates and 5% for those with a college degree or above (Organisation for Economic Co-operation and Development [OECD], 2013b, p. 21). Further, 30% of those who reported being out of the workforce took the paper-based test as opposed to 14% for adults in the labor force (Organisation for Economic Co-operation and Development [OECD], 2013b, p. 21), suggesting a correlation between skills required for completing the computerized version of the assessment and employability (Vanek, 2017).

An issue that PIAAC attempts to provide a clear picture for is the match between supply and demand for employment skills (Organisation for Economic Co-operation and Development [OECD], 2016, p. 3). There has been increasing interest in exploring the relationship between proficiency levels and subgroups by employment-related variables, such as employment status and skills used at work (e.g., Organisation for Economic Co-operation and Development [OECD], 2016, p. 102–103; Perry et al., 2016). However, assessment of skills is merely one step toward a more balanced labor market. Knowing which subgroups performed better is a good starting point, but the processes that gave rise to the final proficiency levels are more informative for providing necessary education.

To bridge the gap between supply and demand and provide targeted intervention, it is important to understand which subgroups performed at a lower level and why. Specifically, how did these respondents arrive at a specific wrong answer, and how did subgroups differ in terms of problem-solving strategies? In this regard, more fine-grained investigation on multiple sources of data is needed, which cannot be easily achieved by utilizing response data alone.

The use of computers as the delivery platform enables data collection not just on whether respondents are able to solve the tasks. It also gives information on how they solved them, which is referred to as process data. Process data has great potential for providing insight into different phases of educational learning. One key application area is allowing intelligent tutoring systems to adapt to respondents’ needs in real time based on their characteristics (e.g., Baker, 2007; D’Mello et al., 2008; Scheuer and McLaren, 2011). Another area that has attracted much interest is to model changes in knowledge over time via Bayesian knowledge tracing (e.g., Corbett and Anderson, 1994; Baker et al., 2008; Pavlik et al., 2009).

More importantly, several studies have revealed the critical role of process data in understanding different problem-solving strategies (e.g., Hurst et al., 1997; Vendlinski and Stevens, 2002; He and von Davier, 2015, 2016; He et al., 2018). Vendlinski and Stevens (2002) identified three strategy levels that students adopted to solve a chemistry item: limited, prolific, and efficient. Students who used a limited strategy tried only a few options before attempting to solve the item, whereas the prolific strategy was to explore almost all options on the menu, similar to the “unfocused problem-solving strategy” found in Hurst et al. (1997). On the contrary, students with efficient strategy concentrated only on the key pieces of information, resulting in the highest probability of a correct answer. He and von Davier (2015) further pointed out that the pattern of robust sequences of actions differed significantly by respondents’ performance levels by respondents’ performance levels, which was found consistently were consistent across countries. Those in the higher-performing group tended to use more tools such as search and sort, had clearer understanding of the subgoals, and were able to recover from initial mistakes. The lower-performing group, however, demonstrated more hesitative behaviors, such as clicking “cancel” repeatedly, and only had a vague idea about the purpose of the item (He and von Davier, 2016). He et al. (2018) continued investigating the differences in problem-solving strategies associated with background variables on one PIAAC item across six countries. It was found that test takers with high levels of skills for using ICT at home were more likely to have higher PSTRE performance. Respondents with different genders had significant differences in digital task-solving strategies. In fact, older people, female, and those with low ICT skill use at home or work showed a need for more intervention to improve their PSTRE skills.

Based on the results from He and von Davier (2015, 2016) and He et al. (2018), the present study mainly focuses on employment-related variables and the United States sample to further identify important factors associated with problem-solving skills. Specifically, two research questions are addressed via exploring the process data from one representative PSTRE item:

(1) Which employment-related background variables are significantly related to performance in the PSTRE, literacy, and numeracy domains in the United States sample?
(2) For those subgroups that showed significantly different performance on a representative PSTRE item, what features can we extract from process data to best characterize their behaviors?

By analyzing process data in different employment situations and with different work experience, we are able to see different behavioral patterns by subgroups during the process of solving digital tasks. The rest of this paper is structured as follows. In Section “Materials and Methods” we elaborate on the data and instrument used in this study, and introduce the proposed approach (i.e., regression analysis and feature identification) to map the background variables with action sequences in process data. The results corresponding to the two research questions are presented in Section “Results”, with special attention to generalizing results for the United States population. In the last section, we summarize the findings and discuss the limitations and potential future work using process data in large-scale assessments.

## Materials and Methods

### Datasets and Instruments

The PSTRE assessment in PIAAC 2012 study included 14 items, with seven in each of the two booklets.^1^ Respondents who responded to the PSTRE items had to have some prior computer experience and to have passed the first two stages of core computer-based assessments. The PSTRE items were generally designed in four different environments—email, web, word processor, and spreadsheet; each item involved one or two environments as summarized in Table 1.

**Table 1:** Summary of environments in each item.

Booklet	Order	Item	Email (MC)	Web (WB)	Word Processor (WP)	Spreadsheet (SS)
PS1	1	U01a	1			
PS1	2	U01b	1			
PS1	3	U03a		1		1
PS1	4	U06a		1		
PS1	5	U06b		1		
PS1	6	U21		1		
PS1	7	U04a	1			1
PS2	1	U19a	1			1
PS2	2	U19b			1	1
PS2	3	U07		1		
PS2	4	U02	1	1	1	
PS2	5	U16	1			
PS2	6	U11b	1			
PS2	7	U23	1	1		

Item U02, the Meeting Room Assignment item, was chosen as an example to illustrate the present study. There are three environments involved in this item: email, web, and word processor. Respondents were asked to read through a list of emails of meeting room requests in the email environment, and then try to fill out as many requests as possible in the room reservation system in a web environment.

There are four reasons why we decided to use U02 as an example:

(1) U02 was rather difficult for United States respondents: 932 (70%) respondents received no credit, 294 (22%) received partial credit, and only 114 (9%) got full credit. Such an item could potentially provide more information to identify reasons for failure when tracking respondents’ process data. Researchers have found that for a moderately difficult item, respondents tend to demonstrate more heterogeneous use of strategies, aberrant response behavior, and response time (e.g., Vendlinski and Stevens, 2002; Goldhammer et al., 2014; de Klerk et al., 2015). To explore the difference between respondents who at least got part of the item correct and those who received no credit, the polytomous scores were dichotomized by collapsing partial credit and full credit in the present study.
(2) U02 had multiple environments (email, web, and word processor), which tended to have more diverse actions from which to extract information.
(3) Compared to items at the beginning or the end, items in the middle of the booklet were less likely to demonstrate position effect (e.g., Wollack et al., 2003).
(4) U02 shared environments with most items in booklet PS2. This provided the possibility to investigate the consistency of problem-solving strategies across items for each individual.

The present study used two datasets, the public-use background questionnaire (BQ) from PIAAC 2012 and the assessment’s log file. The former dataset contains the original and derived variables from the BQ, cognitive response data, as well as sampling weights. The employment-related variables reflected different perspectives of the test taker’s employment situation, such as employed or not, whether the test taker had a supervisor role, related work experience, computer use at work, and so on. The demographic variables included age, gender, the test taker’s education level, the test taker’s parents’ education level, whether the assessment was given in the test taker’s native language, and the number of books at home. Variables from the BQ, with a focus on those related to employment and work experience, were used to explore the relationship between patterns extracted from process data and respondents’ employment situations. Variables measuring skills used at home, such as ICT and numeracy skills at home, were not considered since work-related background variables had stronger connections to employment situation (Organisation for Economic Co-operation and Development [OECD], 2016).

Additionally, scored responses, total response time, timing of first action, and number of actions were available for each item in the three domains. For each of the 3 domains, 10 plausible values were provided for each test taker (see more information in Organisation for Economic Co-operation and Development [OECD], 2013a, Chapter 17). The proposed analyses were conducted with and without sampling weights, and the differences were marginal. Therefore, we reported results with sampling weights only. Log files recorded the actions taken during the assessment, including actions taken during the assessment, such as sorting, clicking menu, opening a folder, using the help function, and so on.

The total sample size for the BQ was 5,010.^2^ The descriptive statistics of age, gender, and education of all respondents in the BQ were reported in Tables 2, 3. The distributions of age and gender are rather even. About 46% of the respondents obtained postsecondary education, 39% had upper secondary education, and 13% had lower secondary education or less.

**Table 2:** Descriptive statistics of age and gender for all respondents in BQ.

Level	Age	Count (%)	Gender	Count (%)
1	24 or less	837 (16.71%)	Male	2,323 (46.37%)
2	25–34	1,045 (20.86%)	Female	2,687 (53.63%)
3	35–44	978 (19.52%)		
4	45–54	1,084 (21.64%)		
5	55 plus	1,066 (21.28%)		

**Table 3:** Descriptive statistics of education level for all respondents in BQ.

Level	Education	Count (%)
1	Lower secondary or less (ISCED 1,2, 3C short or less)	629 (12.55%)
2	Upper secondary (ISCED 3A-B, C long)	1,977 (39.46%)
3	Postsecondary, non-tertiary (ISCED 4A-B-C)	394 (7.86%)
4	Postsecondary, tertiary – professional degree (ISCED 5B)	414 (8.26%)
5	Postsecondary, tertiary – bachelor degree (ISCED 5A)	902 (18.00%)
6	Postsecondary, tertiary – master/research degree (ISCED 5A/6)	578 (11.54%)
7	Missing	116 (2.32%)

### Data Analyses

**FIGURE 1:**
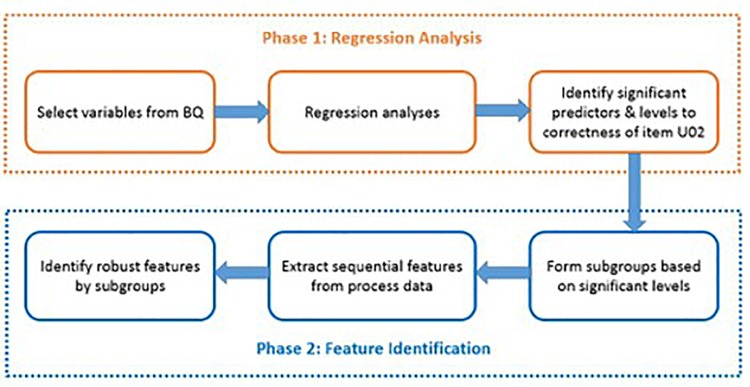
An overview of the two-phase analysis.

The present study was conducted in two phases: regression phase and feature identification phase (see Figure 1 as an overview). In the first phase, we employed regression analyses to select background variables that could significantly predict respondents’ PSTRE, literacy, and numeracy proficiency levels, response time, as well as response correctness in the example item. In the second phase, typical action sequences were identified by different subgroups using the chi-square feature selection model.

#### Regression Phase: Identifying Significant Employment-Related Background Variables

Regression analyses were conducted to examine which employment-related variables have significant associations with both person- and item-related outcome variables. The variables were carefully selected from the BQ, including 20 employment-related and 6 demographic variables. Table 4 summarizes the description, number of non-missing categories, and the reference category for each variable (see Appendix Table A1 for detailed descriptions for all levels of each variable). To avoid a dramatic decrease in sample size and incorporate information as much as possible in the regression analysis, we coded missing responses in the selected variables as an additional category and retained in the regression analyses. This method was popularized by Cohen and Cohen (1985) as a way to deal with missing responses in categorical variables. This method incorporates all the available information into the regression analyses, whereas other methods could heavily depend on data collection design and model specification (e.g., Howell, 2008). Compared to the deletion methods, the generalizability of the results to the United States population may also be retained using the proposed method. Moreover, it is the simplest approach to addressing missing data with some missing mechanisms being untestable (Carpenter and Goldstein, 2004; Horton and Kleinman, 2007).

**Table 4:** Summary of BQ variables used in the present study.

No.	Variable	Description	Number of categories	Reference category
(1)	PAIDWORK^a^	Incidence of paid work experience	4	1. No work ever
(2)	C_D05	Employed/unemployed/out of labor force	3	1. Employed
(3)	D_Q04_T1^b^	Is an employee/supervisor/ self-employed/unpaid	4	1. Employee, not supervisor
(4)	D_Q08b	Managing how many employees	5	1. 1–5 people
(5)	D_Q12b	Education level sufficient/too high/too low to do job satisfactorily	3	1. Necessary
(6)	D_Q12c_RC^c^	Related work experience in years	4	1. No experience
(7)	F_Q05a	Incidence of solving simple problems	5	1. Never
(8)	F_Q05b	Incidence of solving complex problems	5	1. Never
(9)	F_Q07b	Need more training for skill use at work or not	2	1. Yes
(10)	G_Q06	Level of computer use	3	1. Straightforward
(11)	ISCOSKIL4	Skilled/semi-skilled/ elementary occupations	4	1. Skilled occupations
(12)	EARNMTHALLDCL	Monthly earning decile including all incomes	10	1. Lowest decile
(13)	LEARNATWORK_WLE_CA	Index of learning at work	6	0. All zero response
(14)	ICTWORK_WLE_CA	Index of use of ICT skills at work	6	0. All zero response
(15)	INFLUENCE_WLE_CA	Index of use of influencing skills at work	6	0. All zero response
(16)	NUMWORK_WLE_CA	Index of use of numeracy skills at work	6	0. All zero response
(17)	PLANNING_WLE_CA	Index of use of planning skills at work	6	0. All zero response
(18)	READWORK_WLE_CA	Index of use of reading skills at work	6	0. All zero response
(19)	TASKDISC_WLE_CA	Index of use of task discretion at work	6	0. All zero response
(20)	WRITWORK_WLE_CA	Index of use of writing skills at work	6	0. All zero response
(21)	AGEG10LFS	Age in 10-year bands	5	1. 24 or less
(22)	PARED	Highest of parents’ level of education	3	1. Neither parent have attained upper secondary
(23)	NATIVELANG	Test language same as native language or not	2	0. Test language is not native language
(24)	EDCAT6	Highest level of formal education obtained	6	1. Lower secondary or less
(25)	GENDER_R	Gender	2	1. Male
(26)	J_Q08	Number of books	6	1. 10 books or less

*^a^PAIDWORK is recoded from NOPAIDWORKEVER (never had paid work), PAIDWORK5 (have had paid work in the last 5 years) and PAIDWORK12 (have had paid work in the last 12 months). ^b^D_Q04_T1 provides information about whether the respondent is an employee, supervisor, self-employed, or unpaid. This variable was chosen since research has shown that the relationship between some variables is different for wage earners and self-employed workers, such as earnings and conscientiousness (Organisation for Economic Co-operation and Development [OECD], 2016). ^c^D_Q12c_RC is recoded from D_Q12c (related work experience in years) by collapsing category 2–4.*

With respect to the dependent variables, we used the respondents’ scores in PSTRE, literacy, and numeracy as well as total response time and binary scores (correct as 1; incorrect as 0) in U02. To retain as much information as possible in the regression analysis, we included all respondents who had plausible values in each domain, resulting in a total sample size of 4,103, 4,898, and 4,898 for PSTRE, literacy, and numeracy scores, respectively. Further, we used total item response time (U02RT) and dichotomized scores (U02score) as item-related variables in the regression analysis for the Meeting Room Assignment item. Note that only 1,340 in the sample who had process data for this specific item were adopted in the current study, occupying one third of the whole sample size used in the regression analysis.

Of the five outcome variables, linear regression was conducted for the four continuous outcome variables—PSTRE, literacy, and numeracy scores, as well as the item response time on the Meeting Room Assignment item. For the dichotomized scores, a logistic regression was carried out. The regression analyses were conducted using the International Association for the Evaluation of Educational Achievement (IEA)’s International Database (IDB) Analyzer version 4.0.16.0 (International Association for the Evaluation of Educational Achievement [IEA], 2013) to interface with SAS 9.4 SAS Institute, 2015). In this study, each regression analysis was carried out using a full sample weight and 45 replicate weights, as well as 10 plausible values if the outcome variable was the scores from one of the three domains. The final regression coefficient estimates were weighted averages of the coefficient estimates from each round. The standard errors of the coefficient estimates were pooled standard errors reflecting variability due to multiple imputation and/or sampling error. Then the significance of the coefficient estimates was determined by the relative magnitude of the final coefficient estimates and the pooled standard errors. Readers can refer to International Association for the Evaluation of Educational Achievement [IEA] (2013) for more information.

#### Feature Identification Phase: Identifying Typical Action Sequences by Subgroups

In the feature identification phase, process data were used to understand the inherent differences among respondents’ action sequences in the test-taking process. Each individual’s time-stamped action sequences in U02 were extracted from the log file and recoded into (mini-) sequences by *n*-grams.

An *n*-gram is defined as a contiguous sequence of *n* words in text mining; similarly, when analyzing action sequences from process data, an *n*-gram can be defined as a sequence of *n* adjacent actions (Manning and Schütze, 1999). For instance, a typical sequence for email review actions is recorded as “*MAIL_VIEWED_4, MAIL_VIEWED_2, MAIL_VIEWED_1*”, the unigram is each of the three separate actions (e.g., “*MAIL_VIEWED_4*”), the bigram is the two adjacent actions as one unit, (e.g., “*MAIL_VIEWED_2, MAIL_VIEWED_1*”), and the trigram is the three adjacent actions as one unit (e.g., “*MAIL_VIEWED_4, MAIL_VIEWED_2, MAIL_VIEWED_1*”). In this study, we focused on unigrams, bigrams, and trigrams, which are adjacent action sequences of length 1, 2, and 3, respectively.

When retrieving information from the *n*-grams, a question regarding whether all terms could be considered equally important based on their raw frequencies needs to be addressed. In fact, certain terms have little or no discriminating power in determining relevance; it was recommended to give them less weight when classifying different subgroups (Manning and Schütze, 1999). We adopted term weights in this study to adjust for between- and within-individual differences in action frequencies. In terms of between-individual differences, a popular weighting method in text mining, inverse document frequency (IDF; Spärck Jones, 1972) that was renamed as inverse sequence frequency (ISF; He and von Davier, 2016) was adapted for estimating the weight of each *n*-gram. ISF is defined as ISF_i_ = log (*N*/*sf_i_*) ≥ 0, where *N* denotes the total number of sequences in the sample, which is the same as the total number of respondents, and *sf_i_* represents the number of sequences containing action, i.e., a large ISF reflects a rare action in the sample, whereas a small ISF represents a frequent one.

Within-individual differences occur when an individual takes some actions more often than others. Although more frequent sequences are usually more important than less frequent sequences, the raw frequencies of these action sequences often overestimate their importance (He and von Davier, 2015, 2016). To account for within-individual differences in the importance of action sequences, a weighting function was employed *f*(*tf*_ij_) = 1 + log (*tf*_ij_), where *tf*_ij_ > 0 represents the frequency of action *i* in sequence *j* (Manning and Schütze, 1999). Combining the between- and within-individual weights, the final action weight can be defined as *weight* = (*i,j*) = [1 + log (*tf*_ij_)] log(*N*/*sf*_i_) for *tf*_ij_ ≥ 1. In contrast to raw frequency, this weighting mechanism was applied for attenuating the effect of actions or action vectors that occurred too often to be meaningful. (For more details of n-grams and term weights in process data analysis, refer to He and von Davier, 2015, 2016).

To answer the question regarding which actions or mini action sequences (i.e., *n*-grams) are the key factors that distinguish subgroups, we applied a commonly used tool in natural language processing—the chi-square feature selection model (Oakes et al., 2001)—to identify robust classifiers. The chi-square feature selection model is recommended for use in textual analysis due to its high effectiveness in finding robust keywords and for testing the similarity between different text corpora (e.g., Manning and Schütze, 1999; He et al., 2012, 2014, 2017). The definition of “robust” is different from what is defined in statistics; here, robust features are generally defined as the “best” features with high information gain in natural language processing (Joachims, 1998). Chi-square scores assigned to the features were ranked in a descending order, and those with the highest scores were defined as robust features. Specifically, frequencies and weights of certain actions for different employment statuses were used as input for the chi-square selection model.

Features extracted for different groups (e.g., income and employment type) were used to understand the inherent differences in typical sequences among subgroups. The package “tm” (Feinerer, 2017) in R version 3.3.3 (R Core Team, 2017) was utilized for applying chi-square selection model to identify robust features. We formed subgroups based on each significant employment-related predictor for the outcome variable U02score (i.e., binary variable correctness/incorrectness in the Meeting Room Assignment item). The significant level of the predictor was compared with the reference level of the predictor. For instance, if the fourth decile of EARNMTHALLDCL was significantly different from the reference group, two subgroups were formed by respondents in the lowest decile and in the fourth decile. Chi-square selection model was then applied to compare action sequences between these two subgroups and identify robust features to distinguish them.

## Results

### Regression Phase

The distributions of the background variables were checked to ensure the representativeness of this sample. The difference between the percentages of each category of the background variables from the sample with valid scores on the U02 item (i.e., Meeting Room Assignment) and the total sample was usually around 1–2% (see Appendix Table A2 for details). As such, we deemed that the differences were not substantially different.

The sample size and descriptive statistics of the five outcome variables—PSTRE, literacy, and numeracy scores, and the response time and dichotomized scores for item U02 —are reported in Table 5. Using scores from all three domains as dependent variables enabled us to explore the uniqueness of PSTRE skills. In other words, which employment-related variables were significant in predicting PSTRE scores but not literacy or numeracy scores. The significant predictors identified from regression analyses are summarized in Table 6 with respect to each of the five outcome variables. Table 7 presents the unstandardized coefficient estimates for the significant variables. The standardized coefficients for all variables were reported in Appendix Table A3, as a measure of variables’ contributions to predicting the outcome that accounts for contributions of other independent variables (e.g., Menard, 1995, 2004; Zientek et al., 2008; Nathans et al., 2012). The rank ordering of the absolute values of these coefficients indicates the relative importance of the variables.

**Table 5:** Sample size and descriptive statistics of the outcome variables.

Variable name	Sample size	Minimum	Maximum	Mean	Standard deviation
PSTRE	4,103	113.56	425.01	277.98	43.11
Literacy	4,898	100.94	424.33	271.84	48.28
Numeracy	4,898	40.33	426.90	254.68	55.80
U02RT^∗^	1,340	5.16	2704.38	215.67	208.63
U02score	1,340	0	1	0.30	0.21

*^∗^U02RT is reported in seconds.*

**Table 6:** Summary of significant predictors.

No.	Variable	Description	PSTRE	Literacy	Numeracy	U02RT	U02score
(1)	PAIDWORK	Incidence of paid work experience		1^∗^	1		
(2)	C_D05	Employed/unemployed/out of labor force					
(3)	D_Q04_T1	Is an employee/supervisor/self-employed/unpaid					
(4)	D_Q08b	Managing how many employees					
(5)	D_Q12b	Education level sufficient/too high/too low to do job satisfactorily	1	1	1		
(6)	D_Q12c_RC	Related work experience in years	1		1		
(7)	F_Q05a	Incidence of solving simple problems	1	1	1		
(8)	F_Q05b	Incidence of solving complex problems		1	1		
(9)	F_Q07b	Need more training for skill use at work or not		1	1		
(10)	G_Q06	Level of computer use	1	1	1	1	
(11)	ISCOSKIL4	Skilled/semi-skilled/elementary occupations	1	1	1		
(12)	EARNMTHALLDCL	Monthly earning decile including all incomes	1	1	1		1
(13)	LEARNATWORK_WLE_CA	Index of learning at work	1	1	1		
(14)	ICTWORK_WLE_CA	Index of use of ICT skills at work	1	1	1		
(15)	INFLUENCE_WLE_CA	Index of use of influencing skills at work		1	1		1
(16)	NUMWORK_WLE_CA	Index of use of numeracy skills at work	1	1	1	1	
(17)	PLANNING_WLE_CA	Index of use of planning skills at work		1	1	1	
(18)	READWORK_WLE_CA	Index of use of reading skills at work	1				1
(19)	TASKDISC_WLE_CA	Index of use of task discretion at work					1
(20)	WRITWORK_WLE_CA	Index of use of writing skills at work		1			1
(21)	AGEG10LFS	Age in 10-year bands	1	1	1		1
(22)	PARED	Highest of parents’ level of education	1	1	1	1	
(23)	NATIVELANG	Test language same as native language or not	1	1	1		1
(24)	EDCAT6	Highest level of formal education obtained	1	1	1	1	1
(25)	GENDER_R	Gender	1		1		
(26)	J_Q08	Number of books	1	1	1		

*^∗^The number 1 indicates that at least one level of this variable was significantly different from 0 at the significance level of 0.05.*

**Table 7:** Summary of unstandardized regression coefficients of significant variables.

Variable	Category	Description	PSTRE	Literacy	Numeracy	U02RT	U02score
Intercept		Intercept	214.93	162.42	126.44	380.70	
PAIDWORK	3	Have had paid work in 5 years but not 12 months		22.37	30.59		
	4	Have had paid work in 12 months			37.51		
D_Q12b	2	A lower education level would be sufficient	5.22	3.76			
	3	A higher education level would be needed	−8.17	−8.38	−9.35		
D_Q12c_RC	2	Less than 1 year of relevant work experience	−5.07		−5.49		
	4	More than 3 years of relevant work experience			6.76		
F_Q05a	2	Solve simple problems less than once a month		9.13			
	3	Solve simple problems less than once a week but at least once a month	13.53	10.91	17.92		
	4	Solve simple problems at least once a week but not everyday	12.58	13.80	17.11		
	5	Solve simple problems everyday	16.47	17.71	19.07		
F_Q05b	5	Solve complex problems every day		−11.01	−9.14		
F_Q07b	2	Do not need more training for skill use at work		6.20	7.28		
G_Q06	2	Moderate level of computer use	9.97	7.23	8.15	39.03	
	3	Complex level of computer use	15.44	10.35	15.96	74.98	
ISCOSKIL4	2	Semi-skilled white-collar occupations	−3.83	−4.47	−4.80		
	3	Semi-skilled blue-collar occupations	−7.29	−7.10	−6.24		
	4	Elementary occupations		−13.49	−14.11		
EARNMTHALLDCL	4	4th decile of monthly earning					2.00
	9	9th decile of monthly earning		7.88			
	10	10th decile of monthly earning	10.15	12.38	11.55		
LEARNATWORK_WLE_CA	4	More than 60–80% on index of learning at work	−7.60	−10.42	−9.74		
ICTWORK_WLE_CA	1	Lowest 20% on index of use of ICT skills at work		10.98	10.05		
	2	More than 20–40% on index of use of ICT skills at work	12.28	15.23	11.28		
	3	More than 40–60% on index of use of ICT skills at work	16.34	13.63	12.09		
	4	More than 60–80% on index of use of ICT skills at work	17.78	11.52	10.29		
	5	More than 80% on index of use of ICT skills at work	20.52	15.46	16.00		
INFLUENCE_WLE_CA	1	Lowest 20% on index of use of influencing skills at work					1.63
	5	More than 80% on index of use of influencing skills at work		−10.84	−10.24		
NUMWORK_WLE_CA	2	More than 20–40% on index of use of numeracy skills at work	9.12	5.70	8.76		
	3	More than 40–60% on index of use of numeracy skills at work			7.10		
	4	More than 60–80% on index of use of numeracy skills at work	8.51	8.33	10.47	50.43	
	5	More than 80% on index of use of numeracy skills at work	6.89		10.26		
PLANNING_WLE_CA	4	More than 60–80% on index of use of planning skills at work		9.33	12.72	55.12	
READWORK_WLE_CA	1	Lowest 20% on index of use of reading skills at work					2.23
	5	More than 80% on index of use of reading skills at work	−14.44				
TASKDISC_WLE_CA	4	More than 60–80% on index of use of task discretion at work					−0.43
WRITWORK_WLE_CA	3	More than 40–60% on index of use of writing skills at work		6.85			
	4	More than 60–80% on index of use of writing skills at work		7.93			1.68
AGEG10LFS	2	25–34	−17.20	−12.91	−12.10		
	3	35–44	−24.57	−17.21	−16.84		
	4	45–54	−31.89	−22.09	−20.77		0.63
	5	55 plus	−35.85	−23.64	−20.65		0.53
PARED	2	At least one parent has attained secondary and postsecondary, non-tertiary	10.31	10.15	13.06	46.16	
	3	At least one parent has attained tertiary	12.33	15.38	15.93	77.01	
NATIVELANG	1	Test language same as native language	13.92	17.41	9.99		1.29
EDCAT6	2	Upper secondary (ISCED 3A-B, C long)	10.09	16.72	21.31	52.29	7.70
	3	Postsecondary, non-tertiary (ISCED 4A-B-C)	14.18	19.91	27.92		7.70
	4	Tertiary – professional degree (ISCED 5B)	17.59	27.84	35.31		5.47
	5	Tertiary – bachelor degree (ISCED 5A)	24.00	35.74	45.59	66.62	10.16
	6	Tertiary – master/research degree (ISCED 5A/6)	28.60	44.53	55.83	79.51	15.18
GENDER_R	2	Female	−4.19		−12.89		
J_Q08	2	11–25 books		6.54			
	3	26–100 books	10.45	9.84	14.52		
	4	101–200 books	13.97	13.54	21.49		
	5	201–500 books	22.49	20.20	26.62		
	6	More than 500 books	14.13	19.74	23.33		

*Coefficients reported in this table for U02score are odds ratios. U02RT is reported in seconds. All numbers shown in the table are significant regression coefficients. The missing cells or missing categories indicate insignificant coefficient values and therefore are not reported. Those cells in gray indicate coefficient estimates that are in consistent with our expectation.*

In general, all five outcome variables had one significant variable in common, EDCAT6, which means that the highest level of formal education is important for obtaining high scores in all three domains and on individual item responses, and it also contributes to longer item response time in this particular item. Among the three person-related dependent variables, more predictors were significant in predicting literacy and numeracy scores when compared with PSTRE scores. The significant variables for literacy and numeracy scores were more similar, though the three domains had 13 significant variables in common. D_Q12c_RC, the related work experience in years, and GENDER_R (gender) were significant in predicting PSTRE and numeracy but not literacy, whereas WRITWORK_WLE_CA (index of use of writing skills at work) was only important for literacy scores. As the focus of this study, PSTRE scores had one unique significant variable—READWORK_WLE_CA (index of use of reading skills at work)—indicating that these skills are significantly related to PSTRE scores. This reflects that by item design, PSTRE items would require higher-level reading skill use at work to understand the item structure, follow the instructions, and browse the website.

Only five variables were significant in predicting the response time on the Meeting Room Assignment item. The regression coefficient estimates showed that respondents who were well-educated, had higher levels of computer use, used more numeracy and planning skills at work, and whose parents also obtained higher education degrees tended to spend more time on the item. Although some research has shown that people with higher ability need less time to finish an item (e.g., van der Linden, 2007; Klein Entink, 2009; Wang and Xu, 2015; Fox and Marianti, 2016), other studies demonstrated the opposite evidence, especially for non-speeded tests (e.g., Roberts and Stankov, 1999; Klein Entink et al., 2009). This observation is consistent with the fact that PIAAC was not a timed assessment; respondents were allowed to take as much time as needed.

Similarly, U02score did not have as many significant variables as the person-related outcomes either (i.e., PSTRE, literacy, and numeracy scores), where only eight variables were significant. It was also noted that not all variables were significant in predicting PSTRE scores. This might be because PSTRE scores are holistic measures of the PSTRE skills, which represent the common construct assessed by the 14 PSTRE items. As U02 only partially contributed to the PSTRE scores, it did not necessarily contain all aspects of the construct.

In terms of the coefficient estimates, most were consistent with our expectations. With respect to employment-related variables, respondents who had paid work, more related work experience, solved simple or complex problems more frequently, had higher level of computer use, had skilled occupations and higher monthly income, and/or had higher index variables tended to have higher scores in the three domains and higher odds of success in this specific item. For the demographic variables, younger male respondents who were well-educated and had many books at home would get higher scores when the test was given in their native languages.

However, some coefficient estimates were inconsistent with our expectations, which are highlighted in gray in Table 7. For example, we would expect respondents with more related work experience to perform better in general, but the estimates for the variable representing experience of less than a year were negative for PSTRE and numeracy scores. This indicates that, controlling for all other variables, having short work experience was not better than having no experience for these two outcomes. For F_Q05b (solve complex problems every day), coefficients for literacy and numeracy scores were also negative when comparing the highest category to the lowest, reflecting that a respondent who solved complex problems regularly might get a score lower than a respondent who never solved complex problems at work. These contradictory results may indicate some interactions among the predictors, which would be worthwhile for further exploration.

### Feature Identification Phase

For the significant predictors for U02score, we further explored how the action sequences of the two groups were different from each other. We used two significant variables—monthly income and education—as concrete examples to show how the features from process data were identified. Given the limited space, we listed more detailed results in the Appendix.

#### Differences by Monthly Income Subgroup

The regression coefficient for the fourth decile of EARNMTHALLDCL (monthly earning decile including all incomes) was significant and positive, indicating that respondents with monthly income in that decile were more likely to get a score of 1 than those in the first (lowest) decile. As such, it is of interest to investigate how the respondents with monthly income in those deciles differed regarding their action sequences. In other words, what features did the two groups of respondents have in their test-taking behaviors that gave rise to higher or lower chances of answering the Meeting Room Assignment item correctly?

As demonstrated earlier, we conducted chi-square selection to identify the most distinguishable *n*-grams between the two groups. Specifically, the top five unigrams, bigrams, and trigrams with the highest chi-square scores were obtained for the focal group and the reference group, respectively. The description and frequency of 34 unigrams used in the present study were reported in Table 8. These robust features were used to understand the most distinctive action sequences between the two groups of respondents. The same procedure was carried out for all significant predictors for U02score. Tables 9, 10 demonstrate monthly income and education as two examples, respectively; the robust features for all the other predictors are reported in Appendix Tables A4–A14 for more details. The interpretations of the actions were based on content experts who designed the item.

**Table 8:** Description and frequency of unigrams.

No.	Features	Description	Frequency
(1)	*FOLDER_VIEWED*	View a folder	5762
(2)	*ENVIRONMENT_WB*	Go to web environment	4715
(3)	*ENVIRONMENT_MC*	Go to email environment	4317
(4)	*MAIL_VIEWED_1*	View 1st email	2725
(5)	*HISTORY_VIEWCALENDAR*	Go to calendar tab in web environment	2190
(6)	*MAIL_VIEWED_3*	View 3rd email	1968
(7)	*HISTORY_RESERVATION*	Go to reservation tab in web environment	1935
(8)	*COMBOBOX_ROOM*	Choose a room when filling out a room request	1891
(9)	*MAIL_VIEWED_4*	View 4th email	1698
(10)	*MAIL_VIEWED_2*	View 2nd email	1544
(11)	*MAIL_MOVE*	Move an email	1499
(12)	*NEXT_INQUIRY*	Go to next item	1371
(13)	*START*	Start item U02	1326
(14)	*COMBOBOX_START_TIME*	Choose start time when filling out a room request	1312
(15)	*COMBOBOX_END_TIME*	Choose end time when filling out a room request	1304
(16)	*COMBOBOX_DEPT*	Choose department when filling out a room request	1296
(17)	*HISTORY_MEETINGROOMS*	Go to meeting room details tab in web environment	1058
(18)	*ENVIRONMENT_WP*	Go to word processor environment	987
(19)	*SUBMIT_RESERVATION_FAILURE*	Submit a reservation request unsuccessfully	987
(20)	*SUBMIT_RESERVATION_SUCCESS*	Submit a reservation request successfully	971
(21)	*HISTORY_UNFILLED*	Go to unfilled tab in the web environment	551
(22)	*SUBMIT_UNFILLED*	Submit an unfilled request	414
(23)	*FOLDER*	Do folder-related actions (i.e., create/delete a folder)	332
(24)	*HISTORY_HOME*	Click on the home button in the web environment	244
(25)	*CHANGE_RESERVATION*	Change an existing reservation	227
(26)	*KEYPRESS*	Type in word processor environment	152
(27)	*REPLY*	Reply an email	118
(28)	*CANCEL*	Click on cancel button	111
(29)	*HELP*	Use help function	87
(30)	*COPY*	Use copy function	42
(31)	*SEARCH*	Use search function	38
(32)	*SORT*	Use sort function	21
(33)	*PASTE*	Use paste function	15
(34)	*BOOKMARK*	Do bookmark-related actions (i.e., add/delete a bookmark)	13

**Table 9:** Top five features of action sequences selected for the 4th and 1st deciles of monthly earning groups.

Group	*N*-gram	Action sequences	Chi-square
4th decile of monthly earning	Unigram	*FOLDER*	39.08
		*CANCEL*	16.54
		*BOOKMARK*	7.44
		*HISTORY_HOME*	4.02
		*HELP*	1.84
	Bigram	*FOLDER_VIEWED, FOLDER*	24.06
		*FOLDER, FOLDER_VIEWED*	22.93
		*FOLDER, FOLDER*	22.67
		*MAIL_VIEWED_1, MAIL_VIEWED_3*	18.51
		*NEXT_INQUIRY, CANCEL*	17.64
	Trigram	*FOLDER, FOLDER_VIEWED, FOLDER_VIEWED*	21.87
		*MAIL_VIEWED_1, MAIL_VIEWED_3, MAIL_VIEWED_4*	19.70
		*FOLDER_VIEWED, FOLDER, FOLDER*	18.67
		*ENVIRONMENT_MC, MAIL_VIEWED_1, MAIL_VIEWED_3*	17.25
		*MAIL_VIEWED_1, MAIL_VIEWED_3, MAIL_VIEWED_3*	16.68
1st decile of monthly earning	Unigram	*SEARCH*	11.72
		*COPY*	10.90
		*KEYPRESS*	10.51
		*PASTE*	5.89
		*HISTORY_VIEWCALENDAR*	2.81
	Bigram	*ENVIRONMENT_WB, COMBOBOX_START_TIME*	15.78
		*COMBOBOX_END_TIME, HISTORY_VIEWCALENDAR*	11.20
		*HISTORY_RESERVATION, HISTORY_VIEWCALENDAR*	10.50
		*COPY, KEYPRESS*	10.16
		*HISTORY_UNFILLED, HISTORY_RESERVATION*	9.79
	Trigram	*HISTORY_VIEWCALENDAR, HISTORY_RESERVATION, HISTORY_VIEWCALENDAR*	20.16
		*MAIL_VIEWED_3, ENVIRONMENT_WB, HISTORY_UNFILLED*	16.25
		*ENVIRONMENT_MC, ENVIRONMENT_WB, ENVIRONMENT_MC*	16.13
		*ENVIRONMENT_MC, ENVIRONMENT_WB, COMBOBOX_START_TIME*	15.87
		*MAIL_VIEWED_4, ENVIRONMENT_WB, ENVIRONMENT_MC*	14.88

Table 9 presents the top five unigrams, bigrams, and trigrams for the respondents falling within the fourth and first (lowest) deciles of monthly earning. Among the unigrams, folder-related actions were found more often in the fourth decile group, such as fold, add, or delete a folder. There were a few folders in the email environment, though respondents were not required to perform any actions on them. The fourth decile group also applied more cancel-related actions, such as cancel sorting, cancel changing reservation, cancel switching to the next item, and so on. Though cancel actions are sometimes considered hesitative behaviors (He and von Davier, 2015), they could also indicate that the fourth decile group tried different options in the menu to figure out what could be done in the environment.

Other actions that the fourth decile group frequently used were actions associated with bookmarks, clicking the home button in the web environment, and help functions. The bookmarks were accessible via the dropdown menu or a button on the menu bar. Using the bookmark actions, respondents could easily access the pages that they considered important or useful. The home button was right next to the bookmark button on the menu bar, which is a convenient way to return to the main page of the web environment. The help functions were designed in both email and web environments. In the email environment, the help function provided information regarding actions taken for an email, for instance, write, reply, forward, or delete an email. In the web environment, the help function offered instructions on the menu bar items, such as home and bookmark. As expected, the fourth decile group appeared to take more exploratory actions to facilitate their problem-solving process compared to the first decile group.

The unigrams commonly adopted by the first decile group were entirely different. The most discriminating features included search, copy, keypress (pressing a key on the keyboard), paste, and click on the view calendar button. The search function was available in both email and web environments. However, the search function was not required to obtain a correct answer to U02, as the information in the two environments was displayed in short text or tables. The copy, keypress, and paste unigrams were used in the word processor environment solely, where respondents could take notes for the time and location of the meeting room requests and compare to the existing schedules. Similar to search, the three functions only existed to aid the synthesis of available information and conflict schedules. For the view calendar button, respondents used it to retrieve the schedules for each meeting room in a certain time period. Respondents were able to see not only the existing reservations, but also the reservations they made for the meeting room requests.

The lower odds of a correct answer to U02 for the first decile group indicated an association between these functions and lower performance in this group. One explanation for this phenomenon could be that the search function and word processor environment were rather redundant for high-performing respondents since they could collect and synthesize information more efficiently. Applying such functions might be a sign that respondents were having difficulty in comprehending or solving U02. Additionally, the view calendar button seemed to suggest that respondents in this group were still in the process of figuring out the purpose of the item instead of working on solving the problem.

Compared to the unigrams, the robust bigrams and trigrams were often more closely related for a certain group. The bigrams for the fourth decile mainly involved folder-related actions, email-viewing actions, and cancel actions; the trigrams also contained similar information. The bigram “*FOLDER, FOLDER_VIEWED*” was found in the trigram “*FOLDER, FOLDER_VIEWED, FOLDER_VIEWED*”; the bigram “*MAIL_VIEWED_1, MAIL_VIEWED_3*” were also included in the robust trigram “*MAIL_VIEWED_1, MAIL_VIEWED_3, MAIL_VIEWED_4*”. This is because bigrams with high frequencies were also likely to appear more commonly when started with or followed by another action. Further, while the five robust unigrams tended to provide unique pieces of information, the five bigrams tended to have overlap due to the increase in sequence length, as did the trigrams. For instance, the top three robust bigrams for the fourth decile group were all folder-related actions, whereas three of the top five trigrams were email-viewing actions.

These mini-sequences of the fourth decile group, along with the unigrams, demonstrated evidence that respondents in this group were working on the item and trying to understand the meeting room requests. It is worth noticing that the emails viewed by the fourth decile group were the first, third, and fourth emails (i.e., *MAIL_VIEWED_1, MAIL_VIEWED_3, MAIL_VIEWED_4*); the second email did not show up in any robust features. In fact, the second email was the only one irrelevant to meeting room requests among the four. Therefore, viewing only the three relevant emails was a strong indication that the respondents at least understood the goal of this item, and were able to filter out emails irrelevant to the goal.

For the first decile group, the respondents did a lot of switching among tabs in the web environment (e.g., *HISTORY_VIEWCALENDAR, HISTORY_RESERVATION, HISTORY_UNFILLED*), or switching among environments (e.g., *ENVIRONMENT_MC, ENVIRONMENT_WB*). Such switching actions indicated that the first decile did not devote much to solving the item. Instead, they seemed to be lost in the item or not interested in exploring more. Results based on unigrams, bigrams, trigrams all suggested that compared to the first decile, respondents in the fourth decile group were more engaged in solving the item. The fourth decile group also adopted more efficient problem-solving strategies, such as bookmark and help. This is consistent with the results from regression analysis that the fourth decile group was more likely to obtain a correct answer to the Meeting Room Assignment item (see Table 7).

#### Differences by Education Subgroups

Another example is the comparison between the robust features from the highest and lowest education groups, as presented in Table 10. Respondents in the highest education group obtained tertiary-master/research degrees, whereas the lowest education group obtained lower secondary education or less. The chi-square selection method also identified highly distinctive features for the two groups. The most discriminating unigrams for the highest education group were sorting, submitting filled reservation or unfilled request (i.e., *SUBMIT_RESERVATION_SUCCESS, UNFILLED_SUBMIT*), and filling out the room and the start time for the request (i.e., *COMBOBOX_ROOM, COMBOBOX_START_TIME*).

**Table 10:** Top five features of action sequences selected for the highest and lowest education groups.

Group	*N*-gram	Action sequences	Chi-square
Tertiary – master/research degree	Unigram	*SORT*	14.04
		*SUBMIT_RESERVATION_SUCCESS*	7.24
		*COMBOBOX_ROOM*	6.88
		*UNFILLED_SUBMIT*	6.82
		*COMBOBOX_START_TIME*	6.22
	Bigram	*COMBOBOX_END_TIME, COMBOBOX_DEPT*	20.36
		*ENVIRONMENT_WB, ENVIRONMENT_MC*	17.06
		*ENVIRONMENT_MC, MAIL_VIEWED_1*	16.84
		*SUBMIT_RESERVATION_SUCCESS, HISTORY_UNFILLED*	16.73
		*HISTORY_MEETINGROOMS, ENVIRONMENT_MC*	16.57
	Trigram	*ENVIRONMENT_MC, MAIL_VIEWED_1, MAIL_VIEWED_2*	26.36
		*HISTORY_RESERVATION, HISTORY_MEETINGROOMS, HISTORY_VIEWCALENDAR*	23.26
		*MAIL_VIEWED_1, MAIL_VIEWED_3, MAIL_VIEWED_4*	20.73
		*COMBOBOX_END_TIME, COMBOBOX_DEPT, SUBMIT_RESERVATION_SUCCESS*	19.96
		*COMBOBOX_START_TIME, COMBOBOX_END_TIME, COMBOBOX_DEPT*	19.35
Lower secondary or less	Unigram	*MAIL_MOVE*	197.12
		*FOLDER_VIEWED*	24.15
		*PASTE*	9.77
		*COPY*	7.73
		*SEARCH*	7.25
	Bigram	*FOLDER_VIEWED, MAIL_MOVE*	159.17
		*MAIL_MOVE, FOLDER_VIEWED*	156.81
		*MAIL_VIEWED_3, MAIL_MOVE*	104.55
		*MAIL_VIEWED_4, MAIL_MOVE*	90.67
		*MAIL_MOVE, MAIL_VIEWED_4*	90.10
	Trigram	*MAIL_MOVE, FOLDER_VIEWED, MAIL_MOVE*	148.51
		*FOLDER_VIEWED, MAIL_MOVE, FOLDER_VIEWED*	95.11
		*MAIL_VIEWED_3, MAIL_MOVE, FOLDER_VIEWED*	92.88
		*FOLDER_VIEWED, MAIL_MOVE, MAIL_VIEWED_3*	92.17
		*MAIL_VIEWED_4, MAIL_MOVE, FOLDER_VIEWED*	86.50

The sorting function was available in the email environment. Respondents could choose to sort by sender, subject, or receiver of the email. Although sorting was not a necessary step to the success of U02, well-educated respondents might consider sorting by subject as a more efficient approach to identifying the emails related to meeting room requests. The *COMBOBOX*-related actions showed evidence of filling out the details of meeting room requests using the dropdown menus. Then, if the requested room and time had no conflict with the existing schedule, one would receive a notice of submitting the reservation successfully. There was also one meeting room request that could not be filled given the current schedule, which needed to be recorded as well. *UNFILLED_SUBMIT* indicated that the test taker also submitted the unfilled request. Such actions were key to the correctness of the Meeting Room Assignment item, because one had to fill out the details of each room request and submit at least one reservation or unfilled request successfully to answer it correctly.

The lowest education group, however, mainly used redundant functions. Moving emails, viewing folder, pasting, copying, and searching were the most important unigrams, which coincidently were found as robust unigrams in the first decile monthly earning group as well. Both the lowest education group and first decile monthly earning group had lower performance on U02 compared with their peers. This finding suggested that lower-performing respondents might be prone to using these unnecessary functions (as defined by content experts), indicating they were unable to figure out a solution.

The robust bigrams and trigrams for the highest education group encompassed some action sequences that also related to filling and submitting the requests, as well as viewing emails, which were required procedures to obtain a correct answer. Some features indicating switching among tabs or environments also appeared. Though we interpreted similar actions for the first decile group as signs of low motivation, these actions could have different meanings for another group. When combined with other robust features for the highest education group, these actions served as connections among necessary steps to finish the item, such as filling in comboboxes and submitting requests. Therefore, the highest education group did not wander around aimlessly, but in fact attempted to synthesize information from multiple environments and make a successful reservation.

Email-moving and folder-viewing actions manifested themselves again in the robust bigrams and trigrams for the lowest education group. These action sequences identified by chi-square selection method demonstrated a clear distinction between the problem-solving processes of the two groups with different education levels. While the highest education group was completing the item with clear subgoals, the lowest education group spent much time and effort moving the emails around and viewing the folder. As a result, these discriminating features identified from the action sequences were in fact strongly associated with the performance on the item.

#### Differences by Other Background Variables

Some general findings from other significant variables resembled the results from the two discussed examples. As presented above, higher income, higher level on the index variables (except for TASKDISC_WLE_CA, index of use of task discretion at work), and higher educational level were associated with higher probability of answering the Meeting Room Assignment item correctly. A younger respondent who took the test in the same language as his or her native language was also more likely to obtain a correct answer. Some background variables have more than one significant dummy variables, such as age and education. It is worth noticing that the features selected for the reference group did not need to be the same when the focal group changed since chi-square chose features that can best distinguish the reference and the focal groups.

Overall, groups with higher odds of a correct answer were likely to adopt the actions related to *SUBMIT* (submitting filled reservation or unfilled request), *COMBOBOX* (filling out the room and the start time for the request), help, and sort. Help and sort are two actions that might be indicative of more efficient problem-solving strategies. To complete the room requests in this item, respondents had to fill time slots for a specific room in the *COMBOBOX* and use one of the two submit buttons. These respondents demonstrated evidence that they went through the necessary steps to obtain correct answers to the Meeting Room Assignment item.

Groups with lower odds of a correct answer, however, used more actions such as *MAIL_MOVE* (moving email) and *SUBMIT_FAILURE* (failure to submit a room request). The occurrence of *MAIL_MOVE* and *SUBMIT_FAILURE* did not always mean that a respondent had trouble finishing an item. A respondent could have been categorizing emails, so he or she could discard those emails that were irrelevant to room requests. If *SUBMIT_FAILURE* was followed by some adjustments in *COMBOBOX* and *SUBMIT_RESERVATION_SUCCESS*, then the respondent made two attempts to submit a reservation and did self-correction. It is when the two actions appeared in the selected features predominantly, and not accompanied by other useful actions, that they might not be able to solve the item.

For some significant dummy variables, *COMBOBOX*-related actions were in fact identified as robust features for the group with lower odds of a correct answer (e.g., INFLUENCE_WLE_CA and READWORK_WLE_CA, or lowest 20% on index of influencing skills at work, and lowest 20% on index of reading skills at work, respectively), while for others, the selected features were mainly associated with *MAIL_MOVE*. Adopting *COMBOBOX*-related actions could be a sign of understanding the purpose of the item and being able to figure out how to fill out the room requests. These respondents were considered closer to the borderline of a correct answer than the group with mostly *MAIL_MOVE* actions and might have had greater potential to get a score of 1 if proper interventions were given. On the contrary, if the majority of a respondent’s actions were *MAIL_MOVE*, he or she might have needed more detailed guidance from the initial steps to submitting the requests.

An intriguing finding is that for the lowest age group (24 or less), the *MAIL_MOVE* action showed up in the top five robust features quite frequently, even though this group was more likely to answer correctly to the Meeting Room Assignment item compared to elder age groups. That is to say, given two respondents with the same occupation, work experience, work-related skills, and so on, the one who was 24 years old or younger would have had a higher probability of a correct response than the one who was 45 to 54, or 55 or older. However, the lowest age group often had different occupations and much less work experience than respondents who were 45 and above. The skills and experiences that the older age groups had accumulated might have enabled them to apply more efficient problem-solving strategies despite younger respondents having more advantage on information technologies. Another possible explanation is that using *MAIL_MOVE* was characteristic of the youngest age group as an action taken without realizing it. They could simply have been moving emails around as they went through the thinking process.

## Discussion

This study aimed at exploring the relationship between sequential problem-solving actions and employment-related variables, and identified the key features for respondents with different levels of employment-related variables. We focused on the data from BQ and log files for the United States population on one representative PSTRE item, the Meeting Room Assignment item, in the main study of 2012 PIAAC. The study was conducted in two phases: (a) use of regression analyses to identify background variables having significant associations with PIAAC performance, and (b) application of chi-square selection method to select robust features of the significantly different groups.

In general, most significant variables and their regression coefficients were consistent with our expectations. Respondents who were well-educated and young, and had more work experience and higher work-related skill use, tended to have higher scores in the three domains and higher odds of success in the Meeting Room Assignment item. Comparing scores in the three domains, the significant variables for literacy and numeracy scores were more similar. PSTRE scores had one unique significant variable—READWORK_WLE_CA (lowest 20% on index of use of reading skills at work)—indicating that PSTRE items might require higher-level reading skill use at work to understand the item structure, follow the instructions, and browse the website.

We further explored the process data to investigate what action sequences were associated with the variables that were significantly related to success in the Meeting Room Assignment item. Based on the final goal of submitting meeting room requests, there were five necessary steps in the problem-solving process for the studied item: (a) read emails; (b) choose the emails related to meeting room requests; (c) synthesize information from multiple environments; (d) determine the requests that could or could not be filled; (e) and submit filled reservations and unfilled requests. Similar to what He and von Davier (2015, 2016) found, respondents who had higher income, work-related skill use, and education level demonstrated clear subgoals in solving the item. For instance, respondents with higher income performed more *MAIL_VIEWED* actions; they were also able to focus on emails directly related to meeting room requests. *SUBMIT* and *COMBOBOX* actions were commonly applied by those with higher work-related skill use at work. Respondents with high education level and high writing skill use at work tended to use more sorting actions.

Some key actions were found more often in the groups with higher income and work-related skill use. Such group were generally prone to adopt *SUBMIT_RESERVATION_SUCCESS, UNFILLED_SUBMIT*, and actions related to *COMBOBOX*, *HELP*, and *SORT*. These actions demonstrated evidence that the respondents went through necessary steps to fulfill room requests in this item. Groups with lower income and work-related skill use, however, took more actions such as *MAIL_MOVE* and *SUBMIT_FAILURE*, which were either redundant or an indication of failing to complete a request.

The most important implication of the present study was that features identified from process data shed light on how much intervention a certain group of respondents might need. There was clear evidence from process data for the steps to read emails, filter the irrelevant email, and submit requests. For instance, respondents who adopted *COMBOBOX*-related actions but still failed to solve the item may have already mastered the majority of required PSTRE skills. Therefore simple instructions on the final steps might be sufficient for them to obtain a correct answer. In contrast, *MAIL_MOVE* and *FOLDER* could be a sign that the respondents needed more comprehensive guidance and training on PSTRE skill. However, evidence for synthesizing information and addressing conflicts were not as traceable. Given sufficient evidence for each required step, further analyses could potentially determine at which specific step an intervention was needed. It also provides the possibility of scoring complex items like PSTRE items base on process data in the future.

Overall, groups with different levels of background variables often demonstrated quite distinctive characteristics with respect to test-taking behaviors. Actions indicative of low PSTRE skill for one group may not mean the same for another group. Therefore, it is important to establish a basic understanding of the common action sequences that a group would take before making decisions on the necessary training and interventions.

When interpreting the robust features identified from process data, it is recommended that one considers unigrams, bigrams, and trigrams simultaneously. This would provide a more holistic view of the respondents’ problem-solving strategies. One example of this is the sequential action of switching among environments. This action could be indicative of aimless behaviors if it was predominant; it could also be the transition among required steps, such as reading emails and submitting requests, if a wide range of features appeared. Therefore, the diversity of the robust features was found informative regarding the interpretation of action sequences.

Despite innovations in this study, at least four limitations are worth mentioning. First, we restricted this study to United States respondents only. Findings related to test-taking behaviors and culture effects that might be learned from other countries were not taken into consideration. Nonetheless, the proposed research plan is applicable to data from other countries. Researchers may compare patterns and action sequences extracted from other countries to those from the United States sample to obtain further insights regarding cross-country differences.

Second, the study focused on process data from the PSTRE domain only. Considering the respondents who had scores in all three domains in the BQ dataset, the correlations between PSTRE scores and literacy/numeracy scores are about 0.81 and 0.76, respectively, for the United States in the 2012 PIAAC assessment (Organisation for Economic Co-operation and Development [OECD], 2013a, p. 7, Chapter 18). Given the strong correlations, the associations between respondents’ sequential action patterns in PSTRE and other domains could be evaluated in future studies.

Third, we used the method suggested by Cohen and Cohen (1985) to deal with missing responses in the BQ, where missing responses were coded as another category for each variable. This method was employed in the present study to retain all available information when missingness occurred in the independent variables (Howell, 2008) and when the missing proportion was higher than 5% or 10% (Schafer, 1999; Bennett, 2001). However, the interpretability of the results becomes a problem (Howell, 2008). Some researchers have also found that this method may produce biased estimates for the regression coefficients under some circumstances, even though it produced reasonably accurate standard error estimates (Jones, 1996; Allison, 2001). Though comparing different approaches to dealing with missing data was not the focus of this study, more advanced methods might be considered in future studies, such as maximum likelihood and multiple imputation (e.g., Bennett, 2001; Howell, 2008).

Lastly, the present study investigated the sequential patterns for different subgroups on only one representative PSTRE item. As the action sequences in process data are highly context dependent (Rupp et al., 2010), the findings from this study need to be cross-validated using other items in a similar context. PSTRE items that share environments with U02 could be further explored to shed light on the consistency of problem-solving strategies across multiple items.

To summarize, this study provides critical evidence of relationships between employment-related background variables and sequential patterns in PSTRE using one example item based on the United States sample in PIAAC. It also provides information to education policy makers to find reasons for success and failure by different employment-related subgroups, thus helping to find an optimal solution to improve their PSTRE skills via a tailored approach. Such information would be key to improving adults’ lifelong learning strategies. Further explorations have been done on multiple items, and similar patterns have been observed, but results were not included to avoid distracting from the main theme of the present study. We recommend to continue exploring the generalizability of results presented in this study across PSTRE items in future studies and to make comparisons across countries and language groups.

## Author Contributions

DL works on forming the research idea, conducting analyses, interpreting results, and write-up of the study. QH works on forming the research idea, interpreting results, and revising the manuscript. HJ works on revising the manuscript, providing suggestions and guidance regarding the overall technical quality of this study.

## Conflict of Interest Statement

The authors declare that the research was conducted in the absence of any commercial or financial relationships that could be construed as a potential conflict of interest.

## Footnotes

1 PSTRE sample items can be found on the National Center for Education Statistics website: https://nces.ed.gov/surveys/piaac/sample_pstre.asp.

2 For the regression analyses in study 1, all respondents with valid PSTRE/literacy/numeracy scores and background variables were used in the analyses. There are two reasons for this. First, it retains the generalizability to the whole United States population. Second, it enables the comparison among the significant variables for the PSTRE, literacy, and numeracy domains to explore the uniqueness of PSTRE skills. Only the sample that responded to PSTRE assessment was used when we further explored features from process data.

## Appendix

**Table A1:** Description of independent variables in the regression analyses.

No.	Variable	Category	Description
(1)	PAIDWORK	1	No work ever
		2	Have had paid work but not in 5 years
		3	Have had paid work in 5 years but not in 12 months
		4	Have had paid work in 12 months
(2)	C_D05	1	Employed
		2	Unemployed
		3	Out of the labor force
(3)	D_Q04_T1	1	Employee, not supervisor
		2	Employee, supervising fewer than 5 people
		3	Employee, supervising more than 5 people
		4	Self-employed or unpaid family worker
(4)	D_Q08b	1	1–5 people
		2	6–10 people
		3	11–24 people
		4	25–99 people
		5	100 or more people
(5)	D_Q12b	1	Current level is necessary
		2	A lower education level would be sufficient
		3	A higher education level would be needed
(6)	D_Q12c_RC	1	No experience
		2	Less than 1 year of relevant work experience
		3	1 or 2 years
		4	More than 3 years of relevant work experience
(7)	F_Q05a	1	Never solve simple problems
		2	Solve simple problems less than once a month
		3	Solve simple problems less than once a week but at least once a month
		4	Solve simple problems at least once a week but not everyday
		5	Solve simple problems everyday
(8)	F_Q05b	1	Never solve complex problems
		2	Solve complex problems less than once a month
		3	Solve complex problems less than once a week but at least once a month
		4	Solve complex problems at least once a week but not everyday
		5	Solve complex problems every day
(9)	F_Q07b	1	Need more training for skill use at work
		2	Do not need more training for skill use at work
(10)	G_Q06	1	Straightforward computer use
		2	Moderate computer use
		3	Complex computer use
(11)	ISCOSKIL4	1	Skilled occupations
		2	Semi-skilled white-collar occupations
		3	Semi-skilled blue-collar occupations
		4	Elementary occupations
(12)	EARNMTHALLDCL	1	1st decile of monthly earning
		2	2nd decile of monthly earning
		3	3rd decile of monthly earning
		4	4th decile of monthly earning
		5	5th decile of monthly earning
		6	6th decile of monthly earning
		7	7th decile of monthly earning
		8	8th decile of monthly earning
		9	9th decile of monthly earning
		10	10th decile of monthly earning
(13)	LEARNATWORK_WLE_CA	0	All zero response
		1	Lowest 20% on index of learning at work
		2	More than 20–40% on index of learning at work
		3	More than 40–60% on index of learning at work
		4	More than 60–80% on index of learning at work
		5	More than 80% on index of learning at work
(14)	ICTWORK_WLE_CA	0	All zero response
		1	Lowest 20% on index of use of ICT skills at work
		2	More than 20–40% on index of use of ICT skills at work
		3	More than 40–60% on index of use of ICT skills at work
		4	More than 60–80% on index of use of ICT skills at work
		5	More than 80% on index of use of ICT skills at work
(15)	INFLUENCE_WLE_CA	0	All zero response
		1	Lowest 20% on index of use of influencing skills at work
		2	More than 20–40% on index of use of influencing skills at work
		3	More than 40–60% on index of use of influencing skills at work
		4	More than 60–80% on index of use of influencing skills at work
		5	More than 80% on index of use of influencing skills at work
(16)	NUMWORK_WLE_CA	0	All zero response
		1	Lowest 20% on index of use of numeracy skills at work
		2	More than 20–40% on index of use of numeracy skills at work
		3	More than 40–60% on index of use of numeracy skills at work
		4	More than 60–80% on index of use of numeracy skills at work
		5	More than 80% on index of use of numeracy skills at work
(17)	PLANNING_WLE_CA	0	All zero response
		1	Lowest 20% on index of use of planning skills at work
		2	More than 20–40% on index of use of planning skills at work
		3	More than 40–60% on index of use of planning skills at work
		4	More than 60–80% on index of use of planning skills at work
		5	More than 80% on index of use of planning skills at work
(18)	READWORK_WLE_CA	0	All zero response
		1	Lowest 20% on index of use of reading skills at work
		2	More than 20–40% on index of use of reading skills at work
		3	More than 40–60% on index of use of reading skills at work
		4	More than 60–80% on index of use of reading skills at work
		5	More than 80% on index of use of reading skills at work
(19)	TASKDISC_WLE_CA	0	All zero response
		1	Lowest 20% on index of use of task discretion at work
		2	More than 20–40% on index of use of task discretion at work
		3	More than 40–60% on index of use of task discretion at work
		4	More than 60–80% on index of use of task discretion at work
		5	More than 80% on index of use of task discretion at work
(20)	WRITWORK_WLE_CA	0	All zero response
		1	Lowest 20% on index of use of writing skills at work
		2	More than 20–40% on index of use of writing skills at work
		3	More than 40–60% on index of use of writing skills at work
		4	More than 60–80% on index of use of writing skills at work
		5	More than 80% on index of use of writing skills at work
(21)	AGEG10LFS	1	24 or less
		2	25–34
		3	35–44
		4	45–54
		5	55 plus
(22)	PARED	1	Neither parent has attained upper secondary
		2	At least one parent has attained secondary and postsecondary, non-tertiary
		3	At least one parent has attained tertiary
(23)	NATIVELANG	0	Test language different from native language
		1	Test language same as native language
(24)	EDCAT6	1	Lower secondary or less (ISCED 1, 2, 3C short or less)
		2	Upper secondary (ISCED 3A-B, C long)
		3	Postsecondary, non-tertiary (ISCED 4A-B-C)
		4	Tertiary – professional degree (ISCED 5B)
		5	Tertiary – bachelor degree (ISCED 5A)
		6	Tertiary – master/research degree (ISCED 5A/6)
(25)	GENDER_R	1	Male
		2	Female
(26)	J_Q08	1	10 books or less
		2	11–25 books
		3	26–100 books
		4	101–200 books
		5	201–500 books
		6	More than 500 books

**Table A2:** Difference in percentages of each category of background variables between the whole sample and the sample with U02 response.

	Levels											
Variables	0	1	2	3	4	5	6	7	8	9	10	Missing
PAIDWORK		1.35	1.42	−0.62	−4.40							2.25
C_D05		−4.86	−1.44	4.08	0.04							2.18
D_Q04_T1		0.13	−1.17	−1.49	−1.81							4.34
D_Q08b		−1.22	0.11	−0.86	−0.51	−0.33						2.81
D_Q12b		−3.28	−0.03	−0.15								3.47
D_Q12c		−0.76	−1.97	−0.46	0.13							
F_Q05a		1.97	0.45	0.38	−2.88	−4.83						4.92
F_Q05b		0.75	−1.02	−1.52	−1.70	−1.38						4.87
F_Q07b		−0.83	−4.05									4.88
G_Q06		−2.61	−4.92	−0.02								7.54
ISCOSKIL4		−5.49	−0.85	0.61	0.73							5.00
EARNMTHALLDCL		−0.44	−0.99	−0.13	−0.74	0.46	−0.32	0.63	−0.19	−1.32	−1.27	4.33
LEARNATWORK_WLE_CA	0.68	0.75	0.09	−2.12	−1.25	−1.54						3.39
ICTWORK_WLE_CA	0.71	−1.79	−0.70	−2.18	−0.95	−2.47						7.38
INFLUENCE_WLE_CA	0.58	1.33	0.21	−2.04	−1.86	−2.88						4.68
NUMWORK_WLE_CA	1.89	−0.91	−1.03	−1.14	−1.32	−2.17						4.68
PLANNING_WLE_CA	1.59	−0.40	−1.29	0.02	−1.40	−3.19						4.68
READWORK_WLE_CA	0.36	0.80	0.78	−1.63	−2.94	−2.05						4.68
TASKDISC_WLE_CA	0.75	0.10	−1.85	−3.27	0.03	−0.48						4.72
WRITWORK_WLE_CA	1.75	0.75	−1.38	−1.65	−1.15	−2.98						4.68
AGEG10LFS		−2.18	−1.34	−1.85	2.08	3.30						
PARED		4.18	−3.25	−4.49								3.57
NATIVELANG	3.05	−5.25										2.20
EDCAT6		3.22	−0.04	−0.79	−0.69	−2.24	−1.63					2.17
GENDER_R		−0.51	0.51									
J_Q08		3.48	−1.41	−1.00	−2.16	−0.97	−0.24					2.29

**Table A3:** Standardized regression coefficients in the regression analyses.

No.	Variable	Category	PSTRE	Literacy	Numeracy	U02RT	U02score
(1)	PAIDWORK	2	0.02	0.02	0.04	0.05	0.46
		3	0.12	0.13^∗^	0.15^∗^	−0.14	0.41
		4	0.12	0.21	0.26^∗^	−0.33	−0.17
(2)	C_D05	2	<0.01	0.13	0.14	0.13	0.17
		3	0.06	0.23	0.26	0.11	0.15
(3)	D_Q04_T1	2	−0.05	0.02	0.02	−0.14	−0.45
		3	−0.11	−0.09	−0.09	<0.01	1.91
		4	−0.09	−0.02	−0.05	<0.01	−0.46
(4)	D_Q08b	2	0.02	0.07	0.07	−0.16	−0.54
		3	0.07	0.07	0.08	−0.12	−0.45
		4	0.04	0.04	0.07	−0.10	−0.38
		5	0.04	0.04	0.05	−0.07	−0.20
(5)	D_Q12b	2	0.05^∗^	0.03^∗^	0.03	0.01	0.03
		3	−0.05^∗^	−0.04^∗^	−0.04^∗^	−0.03	−0.05
(6)	D_Q12c_RC	2	−0.04^∗^	−0.03	−0.04^∗^	−0.02	−0.14
		3	<0.01	<0.01	−0.01	<0.01	<0.01
		4	<0.01	0.02	0.04^∗^	0.04	<0.01
(7)	F_Q05a	2	0.02	0.04^∗^	0.04	0.03	0.41
		3	0.08^∗^	0.06^∗^	0.08^∗^	0.04	0.37
		4	0.12^∗^	0.11^∗^	0.12^∗^	0.04	0.76
		5	0.19^∗^	0.18^∗^	0.17^∗^	0.12	0.90
(8)	F_Q05b	2	<0.01	−0.02	<0.01	−0.01	−0.63
		3	<0.01	−0.03	−0.02	−0.01	−0.53
		4	−0.01	−0.02	<0.01	<0.01	−0.61
		5	−0.02	−0.07^∗^	−0.05^∗^	0.03	−0.44
(9)	F_Q07b	2	0.04	0.06^∗^	0.06^∗^	−0.02	0.04
(10)	G_Q06	2	0.11^∗^	0.07^∗^	0.07^∗^	0.09^∗^	0.64
		3	0.08^∗^	0.05^∗^	0.06^∗^	0.08^∗^	0.33
(11)	ISCOSKIL4	2	−0.04^∗^	−0.04^∗^	−0.04^∗^	−0.01	0.09
		3	−0.06^∗^	−0.05^∗^	−0.04^∗^	−0.02	0.01
		4	−0.03	−0.07^∗^	−0.07^∗^	−0.03	<0.01
(12)	EARNMTHALLDCL	2	−0.02	<0.01	<0.01	−0.06	<0.01
		3	−0.04	−0.04	−0.03	−0.06	−0.07
		4	−0.01	−0.01	<0.01	<0.01	0.09^∗^
		5	−0.03	<0.01	<0.01	−0.06	−0.07
		6	−0.02	<0.01	<0.01	−0.08	−0.03
		7	<0.01	0.03	0.03	−0.06	<0.01
		8	<0.01	0.02	0.02	−0.06	<0.01
		9	0.02	0.04^∗^	0.03	−0.08	0.02
		10	0.06^∗^	0.06^∗^	0.05^∗^	−0.05	0.04
(13)	LEARNATWORK_WLE_CA	1	<0.01	<0.01	<0.01	<0.01	<0.01
		2	<0.01	<0.01	<0.01	−0.03	0.07
		3	−0.03	−0.01	<0.01	−0.02	−0.02
		4	−0.04	−0.02	−0.02	−0.03	−0.03
		5	−0.07^∗^	−0.08^∗^	−0.07^∗^	−0.05	−0.03
(14)	ICTWORK_WLE_CA	1	0.05	0.07^∗^	0.06^∗^	0.01	−0.28
		2	0.09^∗^	0.09^∗^	0.06^∗^	<0.01	−0.20
		3	0.12^∗^	0.08^∗^	0.06^∗^	0.10	−0.16
		4	0.13^∗^	0.07^∗^	0.06^∗^	0.07	−0.10
		5	0.17^∗^	0.11^∗^	0.10^∗^	0.05	−0.15
(15)	INFLUENCE_WLE_CA	1	0.04	−0.01	<0.01	0.02	0.08^∗^
		2	0.06	−0.02	<0.01	<0.01	<0.01
		3	0.03	−0.04	−0.02	<0.01	<0.01
		4	0.03	−0.04	−0.04	−0.06	−0.04
		5	0.03	−0.09^∗^	−0.07^∗^	−0.06	−0.05
(16)	NUMWORK_WLE_CA	1	0.04	0.01	0.01	0.04	<0.01
		2	0.07^∗^	0.04^∗^	0.05^∗^	0.04	<0.01
		3	0.04	0.03	0.04^∗^	0.06	<0.01
		4	0.08^∗^	0.06^∗^	0.07^∗^	0.09^∗^	<0.01
		5	0.06^∗^	0.03	0.07^∗^	0.06	<0.01
(17)	PLANNING_WLE_CA	1	<0.01	0.04	0.04	0.05	−0.03
		2	<0.01	0.01	0.03	0.03	<0.01
		3	<0.01	0.02	0.03	0.05	<0.01
		4	0.03	0.07^∗^	0.08^∗^	0.10^∗^	0.04
		5	<0.01	0.04	0.04	0.05	−0.03
(18)	READWORK_WLE_CA	1	0.02	0.03	0.02	0.06	0.14^∗^
		2	<0.01	0.02	0.02	0.02	0.04
		3	−0.05	0.01	0.01	<0.01	−0.07
		4	−0.07	<0.01	<0.01	0.05	−0.02
		5	−0.14^∗^	−0.02	−0.02	0.04	−0.09
(19)	TASKDISC_WLE_CA	1	−0.02	0.03	0.05	−0.05	<0.01
		2	<0.01	0.05	0.05	−0.09	−0.03
		3	<0.01	0.06	0.05	−0.11	−0.09
		4	<0.01	0.05	0.02	−0.07	−0.15^∗^
		5	0.01	0.05	0.02	−0.11	−0.05
(20)	WRITWORK_WLE_CA	1	<0.01	0.02	0.01	−0.05	−0.10
		2	0.01	0.04	0.02	<0.01	<0.01
		3	<0.01	0.05^∗^	0.03	<0.01	<0.01
		4	0.03	0.06^∗^	0.04	<0.01	0.10^∗^
		5	<0.01	<0.01	−0.03	−0.04	0.03
(21)	AGEG10LFS	2	−0.16^∗^	−0.11^∗^	−0.09^∗^	−0.07	0.05
		3	−0.23^∗^	−0.14^∗^	−0.12^∗^	−0.05	0.01
		4	−0.30^∗^	−0.19^∗^	−0.15^∗^	−0.04	−0.10^∗^
		5	−0.31^∗^	−0.19^∗^	−0.14^∗^	0.02	−0.13^∗^
(22)	PARED	2	0.12^∗^	0.10^∗^	0.11^∗^	0.11^∗^	0.05
		3	0.14^∗^	0.15^∗^	0.13^∗^	0.18^∗^	0.09
(23)	NATIVELANG	1	0.10^∗^	0.13^∗^	0.06^∗^	0.05	0.04^∗^
(24)	EDCAT6	2	0.11^∗^	0.17^∗^	0.18^∗^	0.12^∗^	0.55^∗^
		3	0.09^∗^	0.11^∗^	0.14^∗^	0.06	0.33^∗^
		4	0.12^∗^	0.16^∗^	0.18^∗^	0.03	0.28^∗^
		5	0.21^∗^	0.27^∗^	0.29^∗^	0.12^∗^	0.49^∗^
		6	0.21^∗^	0.28^∗^	0.30^∗^	0.12^∗^	0.48^∗^
(25)	GENDER_R	2	−0.05^∗^	<0.01	−0.11^∗^	<0.01	−0.03
(26)	J_Q08	2	0.04	0.05^∗^	0.02	−0.03	0.22
		3	0.11^∗^	0.09^∗^	0.12^∗^	<0.01	0.29
		4	0.12^∗^	0.1^∗^	0.14^∗^	−0.04	0.27
		5	0.16^∗^	0.12^∗^	0.14^∗^	0.04	0.30
		6	0.08^∗^	0.09^∗^	0.09^∗^	<0.01	0.20

*Significant variables are marked with asterisks. As the logit of the probability of U02score = 1 could range from negative to positive infinity, coefficients for U02score were standardized usingπ/*$\sqrt{3}$*as an approximate for the standard deviation of the dependent variable, as implemented in SAS (e.g., Menard, 1995, 2004).*

**Table A4:** Top five features of action sequences selected for the groups with lowest 20% or all zero response on INFLUENCE_WLE_CA.

Group	*N*-gram	Action sequences	Chi-square
Lowest 20% on	Unigram	*COPY*	6.46
INFLUENCE_WLE_CA		*REPLY*	5.01
		*SEARCH*	4.56
		*HELP*	3.38
		*PASTE*	3.18
	Bigram	*SUBMIT_RESERVATION_SUCCESS, HISTORY_MEETINGROOMS*	11.12
		*UNFILLED_SUBMIT, ENVIRONMENT_MC*	8.10
		*MAIL_VIEWED_3, FOLDER_VIEWED*	7.31
		*HISTORY_VIEWCALENDAR, COMBOBOX_ROOM*	6.77
		*ENVIRONMENT_WB, UNFILLED_SUBMIT*	6.56
	Trigram	*COMBOBOX_DEPT, ENVIRONMENT_MC, ENVIRONMENT_WB*	12.09
		*SUBMIT_RESERVATION_SUCCESS, HISTORY_MEETINGROOMS, HISTORY_VIEWCALENDAR*	9.62
		*HISTORY_UNFILLED, UNFILLED_SUBMIT, ENVIRONMENT_MC*	9.58
		*COMBOBOX_DEPT, SUBMIT_RESERVATION_SUCCESS, HISTORY_MEETINGROOMS*	9.42
		*ENVIRONMENT_MC, ENVIRONMENT_WB, ENVIRONMENT_MC*	9.20
All zero response on	Unigram	*MAIL_MOVE*	17.83
INFLUENCE_WLE_CA		*FOLDER_VIEWED*	9.48
		*CANCEL*	7.97
		*SUBMIT_RESERVATION_FAILURE*	1.58
		*HISTORY_HOME*	0.54
	Bigram	*SUBMIT_RESERVATION_SUCCESS, SUBMIT_RESERVATION_FAILURE*	29.75
		*MAIL_MOVE, FOLDER_VIEWED*	23.45
		*ENVIRONMENT_WB, HISTORY_HOME*	21.24
		*MAIL_VIEWED_3, MAIL_MOVE*	20.17
		*MAIL_VIEWED_2, MAIL_MOVE*	18.77
	Trigram	*FOLDER_VIEWED, MAIL_VIEWED_1, MAIL_MOVE*	30.13
		*MAIL_MOVE, FOLDER_VIEWED, FOLDER_VIEWED*	23.99
		*ENVIRONMENT_WB, COMBOBOX_ROOM, COMBOBOX_START_TIME*	23.23
		*COMBOBOX_DEPT, SUBMIT_RESERVATION_SUCCESS, SUBMIT_RESERVATION_FAILURE*	22.58
		*ENVIRONMENT_MC, NEXT_INQUIRY, CANCEL*	22.00

**Table A5:** Top five features of action sequences selected for the groups with lowest 20% or all zero response on READWORK_WLE_CA.

Group	*N*-gram	Action sequences	Chi-square
Lowest 20% on	Unigram	*HELP*	4.29
READWORK_WLE_CA		*CANCEL*	3.69
		*COPY*	3.59
		*HISTORY_VIEWCALENDAR*	3.28
		*SEARCH*	2.00
	Bigram	*ENVIRONMENT_MC, MAIL_VIEWED_3*	9.10
		*HISTORY_VIEWCALENDAR, HISTORY_UNFILLED*	5.45
		*HISTORY_RESERVATION, COMBOBOX_ROOM*	5.41
		*ENVIRONMENT_WB, HISTORY_VIEWCALENDAR*	5.15
		*CHANGE_RESERVATION, CHANGE_RESERVATION*	4.58
	Trigram	*ENVIRONMENT_MC, ENVIRONMENT_WB, HISTORY_VIEWCALENDAR*	6.70
		*ENVIRONMENT_WB, HISTORY_RESERVATION, HISTORY_VIEWCALENDAR*	6.59
		*ENVIRONMENT_WB, HISTORY_RESERVATION, COMBOBOX_ROOM*	6.27
		*ENVIRONMENT_MC, MAIL_VIEWED_3, ENVIRONMENT_WB*	6.06
		*START, MAIL_VIEWED_1, MAIL_VIEWED_2*	5.64
All zero response on	Unigram	*MAIL_MOVE*	28.76
READWORK_WLE_CA		*FOLDER_VIEWED*	4.66
		*SUBMIT_RESERVATION_FAILURE*	4.58
		*HISTORY_HOME*	4.45
		*REPLY*	1.07
	Bigram	*HISTORY_UNFILLED, HISTORY_UNFILLED*	35.28
		*MAIL_VIEWED_3, ENVIRONMENT_MC*	26.10
		*FOLDER_VIEWED, MAIL_MOVE*	22.78
		*MAIL_MOVE, FOLDER_VIEWED*	22.25
		*HISTORY_HOME, HISTORY_MEETINGROOMS*	20.55
	Trigram	*ENVIRONMENT_WB, COMBOBOX_END_TIME, COMBOBOX_DEPT*	47.51
		*FOLDER_VIEWED, MAIL_MOVE, FOLDER_VIEWED*	35.91
		*MAIL_VIEWED_3, MAIL_MOVE, MAIL_VIEWED_2*	34.97
		*SUBMIT_RESERVATION_FAILURE, COMBOBOX_DEPT, SUBMIT_RESERVATION_FAILURE*	33.24
		*COMBOBOX_ROOM, SUBMIT_RESERVATION_FAILURE, COMBOBOX_DEPT*	33.24

**Table A6:** Top five features of action sequences selected for the groups with 60–80% or all zero response on TASKDISC_WLE_CA.

Group	*N*-gram	Action sequences	Chi-square
60–80% on TASKDISC_WLE_CA	Unigram	*FOLDER*	2.17
		*HISTORY_HOME*	1.87
		*KEYPRESS*	1.52
		*HELP*	0.88
		*MAIL_MOVE*	0.63
	Bigram	*HISTORY_RESERVATION, HISTORY_VIEWCALENDAR*	2.58
		*COMBOBOX_END_TIME, ENVIRONMENT_MC*	2.47
		*MAIL_VIEWED_4, MAIL_VIEWED_3*	2.13
		*ENVIRONMENT_WB, ENVIRONMENT_WP*	1.94
		*ENVIRONMENT_WP, ENVIRONMENT_MC*	1.91
	Trigram	*COMBOBOX_START_TIME, COMBOBOX_END_TIME, ENVIRONMENT_MC*	2.16
		*ENVIRONMENT_MC, ENVIRONMENT_WB, COMBOBOX_DEPT*	2.15
		*COMBOBOX_END_TIME, ENVIRONMENT_MC, ENVIRONMENT_WB*	1.74
		*ENVIRONMENT_WB, COMBOBOX_DEPT, SUBMIT_RESERVATION_SUCCESS*	1.60
		*ENVIRONMENT_WB, HISTORY_RESERVATION, HISTORY_VIEWCALENDAR*	1.44
All zero response on TASKDISC_WLE_CA	Unigram	*SUBMIT_RESERVATION_FAILURE*	3.94
		*COMBOBOX_DEPT*	0.69
		*CHANGE_RESERVATION*	0.41
		*ENVIRONMENT_WP*	0.19
		*COMBOBOX_END_TIME*	0.18
	Bigram	*CANCEL, HISTORY_MEETINGROOMS*	78.31
		*ENVIRONMENT_WB, COMBOBOX_END_TIME*	75.33
		*COMBOBOX_DEPT, COMBOBOX_DEPT*	34.89
		*COMBOBOX_ROOM, ENVIRONMENT_MC*	30.89
		*START, NEXT_INQUIRY*	30.31
	Trigram	*ENVIRONMENT_WB, COMBOBOX_END_TIME, COMBOBOX_DEPT*	138.78
		*MAIL_VIEWED_4, MAIL_VIEWED_4, NEXT_INQUIRY*	96.79
		*ENVIRONMENT_WP, ENVIRONMENT_WB, COMBOBOX_END_TIME*	95.64
		*HISTORY_RESERVATION, COMBOBOX_START_TIME, ENVIRONMENT_MC*	95.64
		*MAIL_VIEWED_1, ENVIRONMENT_WP, ENVIRONMENT_WP*	95.64

**Table A7:** Top five features of action sequences selected for the groups with 60–80% or all zero response on WRITWORK_WLE_CA.

Group	*N*-gram	Action sequences	Chi-square
60–80% on WRITWORK_WLE_CA	Unigram	*CANCEL*	12.84
		*SORT*	12.34
		*BOOKMARK*	5.55
		*UNFILLED_SUBMIT*	5.41
		*SEARCH*	4.88
	Bigram	*SUBMIT_RESERVATION_SUCCESS, COMBOBOX_ROOM*	16.28
		*COMBOBOX_END_TIME, COMBOBOX_DEPT*	15.44
		*ENVIRONMENT_WB, UNFILLED_SUBMIT*	14.59
		*COMBOBOX_END_TIME, SUBMIT_RESERVATION_SUCCESS*	13.67
		*CHANGE_RESERVATION, ENVIRONMENT_MC*	12.38
	Trigram	*ENVIRONMENT_WB, HISTORY_VIEWCALENDAR, HISTORY_RESERVATION*	17.00
		*HISTORY_VIEWCALENDAR, ENVIRONMENT_MC, NEXT_INQUIRY*	16.99
		*MAIL_VIEWED_4, ENVIRONMENT_WB, HISTORY_VIEWCALENDAR*	16.20
		*MAIL_VIEWED_1, MAIL_VIEWED_1, ENVIRONMENT_WB*	15.83
		*ENVIRONMENT_WB, ENVIRONMENT_MC, FOLDER_VIEWED*	15.70
All zero response on WRITWORK_WLE_CA	Unigram	*MAIL_MOVE*	121.84
		*FOLDER_VIEWED*	12.97
		*COPY*	11.99
		*PASTE*	2.66
		*MAIL_VIEWED_2*	1.97
	Bigram	*MAIL_MOVE, FOLDER_VIEWED*	91.26
		*FOLDER_VIEWED, MAIL_MOVE*	89.43
		*MAIL_MOVE, MAIL_VIEWED_1*	74.40
		*MAIL_VIEWED_4, MAIL_MOVE*	72.36
		*MAIL_MOVE, MAIL_VIEWED_3*	69.92
	Trigram	*FOLDER_VIEWED, MAIL_MOVE, MAIL_VIEWED_1*	88.62
		*MAIL_VIEWED_2, MAIL_VIEWED_2, MAIL_VIEWED_2*	81.90
		*MAIL_MOVE, FOLDER_VIEWED, MAIL_MOVE*	76.13
		*MAIL_MOVE, MAIL_VIEWED_1, MAIL_MOVE*	68.14
		*MAIL_MOVE, MAIL_VIEWED_4, MAIL_MOVE*	59.57

**Table A8:** Top five features of action sequences selected for the groups with age 45–54 and age 24 or less.

Group	*N*-gram	Action sequences	Chi-square
Age 45–54	Unigram	*HISTORY_HOME*	71.57
		*HELP*	51.53
		*FOLDER*	44.96
		*REPLY*	31.92
		*COPY*	11.36
	Bigram	*FOLDER_VIEWED, ENVIRONMENT_MC*	61.98
		*HISTORY_HOME, HISTORY_HOME*	56.88
		*HELP, FOLDER_VIEWED*	46.19
		*FOLDER, FOLDER_VIEWED*	42.16
		*START, NEXT_INQUIRY*	41.40
	Trigram	*FOLDER_VIEWED, FOLDER, FOLDER_VIEWED*	49.10
		*HISTORY_HOME, HISTORY_HOME, ENVIRONMENT_MC*	46.57
		*MAIL_VIEWED_3, MAIL_VIEWED_4, ENVIRONMENT_WB*	42.33
		*FOLDER_VIEWED, FOLDER_VIEWED, ENVIRONMENT_MC*	41.07
		*MAIL_VIEWED_1, MAIL_VIEWED_3, MAIL_VIEWED_4*	40.06
Age 24 or less	Unigram	*MAIL_MOVE*	32.37
		*HISTORY_UNFILLED*	11.04
		*CHANGE_RESERVATION*	6.10
		*SUBMIT_RESERVATION_FAILURE*	3.41
		*UNFILLED_SUBMIT*	2.96
	Bigram	*MAIL_VIEWED_3, MAIL_MOVE*	56.40
		*MAIL_VIEWED_1, MAIL_MOVE*	41.25
		*MAIL_MOVE, MAIL_VIEWED_2*	40.61
		*MAIL_MOVE, MAIL_VIEWED_4*	38.06
		*MAIL_VIEWED_4, MAIL_MOVE*	37.37
	Trigram	*START, MAIL_VIEWED_1, MAIL_MOVE*	54.83
		*MAIL_VIEWED_3, MAIL_MOVE, FOLDER_VIEWED*	51.72
		*MAIL_MOVE, MAIL_VIEWED_4, MAIL_MOVE*	43.26
		*MAIL_VIEWED_2, MAIL_VIEWED_2, MAIL_VIEWED_2*	43.11
		*MAIL_VIEWED_1, MAIL_MOVE, FOLDER_VIEWED*	42.37

**Table A9:** Top five features of action sequences selected for the groups with age 55 or more and age 24 or less.

Group	*N*-gram	Action sequences	Chi-square
Age 55 or more	Unigram	*HISTORY_HOME*	95.57
		*HELP*	69.64
		*REPLY*	56.68
		*FOLDER*	53.56
		*SORT*	22.21
	Bigram	*HISTORY_HOME, HISTORY_HOME*	103.26
		*FOLDER_VIEWED, ENVIRONMENT_MC*	84.40
		*FOLDER_VIEWED, FOLDER*	52.83
		*HELP, FOLDER_VIEWED*	48.34
		*FOLDER_VIEWED, REPLY*	39.27
	Trigram	*HISTORY_HOME, HISTORY_HOME, HISTORY_HOME*	75.62
		*FOLDER_VIEWED, FOLDER_VIEWED, ENVIRONMENT_MC*	60.11
		*FOLDER_VIEWED, FOLDER_VIEWED, FOLDER*	41.40
		*FOLDER_VIEWED, ENVIRONMENT_MC, ENVIRONMENT_MC*	39.70
		*HELP, FOLDER_VIEWED, FOLDER_VIEWED*	37.36
Age 24 or less	Unigram	*MAIL_MOVE*	40.37
		*PASTE*	10.44
		*COPY*	6.60
		*ENVIRONMENT_WB*	4.88
		*HISTORY_MEETINGROOMS*	3.82
	Bigram	*MAIL_MOVE, MAIL_VIEWED_2*	43.08
		*FOLDER_VIEWED, MAIL_MOVE*	34.00
		*MAIL_VIEWED_1, MAIL_MOVE*	32.85
		*MAIL_MOVE, MAIL_VIEWED_3*	31.82
		*MAIL_MOVE, FOLDER_VIEWED*	29.20
	Trigram	*MAIL_MOVE, MAIL_VIEWED_2, FOLDER_VIEWED*	34.83
		*ENVIRONMENT_MC, ENVIRONMENT_WB, COMBOBOX_DEPT*	33.78
		*MAIL_VIEWED_2, FOLDER_VIEWED, MAIL_MOVE*	33.19
		*MAIL_MOVE, MAIL_VIEWED_1, MAIL_MOVE*	32.86
		*ENVIRONMENT_WB, COMBOBOX_DEPT, ENVIRONMENT_MC*	28.72

**Table A10:** Top five features of action sequences selected for the groups with test language same as native language or not same as native language.

Group	*N*-gram	Action sequences	Chi-square
Test language same as native language	Unigram	*SORT*	5.64
		*PASTE*	4.37
		*HELP*	3.00
		*UNFILLED_SUBMIT*	2.77
		*FOLDER*	2.29
	Bigram	*HISTORY_VIEWCALENDAR, ENVIRONMENT_WP*	17.51
		*SUBMIT_RESERVATION_FAILURE, HISTORY_VIEWCALENDAR*	10.59
		*FOLDER, FOLDER_VIEWED*	8.49
		*MAIL_VIEWED_3, NEXT_INQUIRY*	8.43
		*COMBOBOX_DEPT, COMBOBOX_START_TIME*	7.32
	Trigram	*ENVIRONMENT_WB, HISTORY_MEETINGROOMS, ENVIRONMENT_MC*	11.37
		*FOLDER_VIEWED, FOLDER_VIEWED, ENVIRONMENT_WB*	10.48
		*ENVIRONMENT_WB, HISTORY_VIEWCALENDAR, HISTORY_UNFILLED*	10.42
		*HISTORY_UNFILLED, ENVIRONMENT_MC, ENVIRONMENT_WB*	10.13
		*HISTORY_VIEWCALENDAR, ENVIRONMENT_WP, ENVIRONMENT_WB*	9.33
Test language not the same as native language	Unigram	*SEARCH*	16.28
		*BOOKMARK*	2.29
		*MAIL_MOVE*	1.16
		*HISTORY_MEETINGROOMS*	0.67
		*COMBOBOX_DEPT*	0.29
	Bigram	*NEXT_INQUIRY, KEYPRESS*	141.04
		*COMBOBOX_ROOM, HISTORY_HOME*	75.69
		*BOOKMARK, HISTORY_VIEWCALENDAR*	67.68
		*HISTORY_RESERVATION, BOOKMARK*	67.68
		*SEARCH, KEYPRESS*	49.98
	Trigram	*HISTORY_HOME, HISTORY_MEETINGROOMS, HISTORY_VIEWCALENDAR*	134.08
		*SUBMIT_RESERVATION_FAILURE, ENVIRONMENT_MC, ENVIRONMENT_WP*	132.41
		*COMBOBOX_END_TIME, COMBOBOX_ROOM, ENVIRONMENT_MC*	130.71
		*FOLDER_VIEWED, SEARCH, SEARCH*	129.22
		*MAIL_VIEWED_2, ENVIRONMENT_WP, ENVIRONMENT_WB*	128.67

**Table A11:** Top five features of action sequences selected for upper secondary and lowest education groups.

Group	*N*-gram	Action sequences	Chi-square
Upper secondary	Unigram	*SUBMIT_RESERVATION_FAILURE*	6.88
		*COMBOBOX_ROOM*	5.08
		*HISTORY_HOME*	5.03
		*COMBOBOX_START_TIME*	3.49
		*BOOKMARK*	3.21
	Bigram	*MAIL_VIEWED_4, MAIL_VIEWED_2*	13.92
		*FOLDER, FOLDER_VIEWED*	13.77
		*HISTORY_HOME, HISTORY_HOME*	13.14
		*FOLDER_VIEWED, FOLDER*	12.18
		*ENVIRONMENT_WB, ENVIRONMENT_MC*	12.15
	Trigram	*ENVIRONMENT_WB, HISTORY_MEETINGROOMS, ENVIRONMENT_MC*	15.66
		*ENVIRONMENT_MC, MAIL_VIEWED_1, MAIL_VIEWED_2*	15.18
		*MAIL_VIEWED_1, MAIL_VIEWED_3, MAIL_VIEWED_4*	14.87
		*HISTORY_RESERVATION, HISTORY_MEETINGROOMS, HISTORY_VIEWCALENDAR*	14.28
		*FOLDER_VIEWED, FOLDER, FOLDER_VIEWED*	13.07
Lower secondary or less	Unigram	*SEARCH*	32.49
		*MAIL_MOVE*	30.71
		*CANCEL*	14.37
		*COPY*	12.53
		*PASTE*	11.72
	Bigram	*SEARCH, FOLDER_VIEWED*	54.79
		*HISTORY_UNFILLED, HISTORY_UNFILLED*	54.12
		*CANCEL, MAIL_MOVE*	53.27
		*CHANGE_RESERVATION, ENVIRONMENT_WP*	52.96
		*MAIL_MOVE, FOLDER*	51.19
	Trigram	*FOLDER_VIEWED, MAIL_MOVE, MAIL_VIEWED_3*	74.80
		*MAIL_VIEWED_4, FOLDER_VIEWED, MAIL_VIEWED_4*	72.11
		*MAIL_MOVE, MAIL_VIEWED_3, MAIL_MOVE*	55.93
		*HISTORY_UNFILLED, HISTORY_RESERVATION, HISTORY_UNFILLED*	52.49
		*MAIL_VIEWED_4, FOLDER_VIEWED, MAIL_VIEWED_1*	52.28

**Table A12:** Top five features of action sequences selected for postsecondary, non-tertiary, and lowest education groups.

Group	*N*-gram	Action sequences	Chi-square
Postsecondary, non-tertiary	Unigram	*HISTORY_HOME*	19.52
		*SORT*	8.20
		*SUBMIT_RESERVATION_FAILURE*	3.59
		*BOOKMARK*	3.29
		*COMBOBOX_ROOM*	1.85
	Bigram	*HISTORY_HOME, HISTORY_HOME*	26.85
		*ENVIRONMENT_WB, ENVIRONMENT_MC*	13.68
		*SUBMIT_RESERVATION_SUCCESS, HISTORY_VIEWCALENDAR*	11.67
		*FOLDER_VIEWED, REPLY*	11.06
		*ENVIRONMENT_MC, MAIL_VIEWED_1*	10.32
	Trigram	*ENVIRONMENT_MC, MAIL_VIEWED_1, MAIL_VIEWED_2*	18.60
		*ENVIRONMENT_WB, ENVIRONMENT_WB, ENVIRONMENT_MC*	18.46
		*ENVIRONMENT_WB, ENVIRONMENT_MC, FOLDER_VIEWED*	16.61
		*ENVIRONMENT_WB, HISTORY_MEETINGROOMS, ENVIRONMENT_MC*	16.19
		*MAIL_VIEWED_1, MAIL_VIEWED_3, MAIL_VIEWED_4*	16.03
Lower secondary or less	Unigram	*SEARCH*	23.04
		*MAIL_MOVE*	19.31
		*PASTE*	6.78
		*HISTORY_UNFILLED*	2.58
		*FOLDER_VIEWED*	2.25
	Bigram	*MAIL_VIEWED_2, MAIL_MOVE*	32.76
		*MAIL_MOVE, MAIL_VIEWED_1*	25.84
		*MAIL_VIEWED_3, MAIL_MOVE*	25.74
		*MAIL_MOVE, MAIL_VIEWED_2*	25.42
		*MAIL_VIEWED_1, MAIL_MOVE*	21.10
	Trigram	*MAIL_MOVE, MAIL_VIEWED_2, MAIL_MOVE*	32.53
		*FOLDER_VIEWED, MAIL_MOVE, MAIL_VIEWED_2*	26.88
		*MAIL_VIEWED_1, MAIL_MOVE, MAIL_VIEWED_1*	23.64
		*MAIL_VIEWED_2, MAIL_MOVE, FOLDER_VIEWED*	23.23
		*MAIL_VIEWED_3, MAIL_MOVE, FOLDER_VIEWED*	21.26

**Table A13:** Top five features of action sequences selected for tertiary-professional degree and lowest education groups.

Group	*N*-gram	Action sequences	Chi-square
Tertiary – professional degree	Unigram	*COMBOBOX_ROOM*	4.48
		*SUBMIT_RESERVATION_SUCCESS*	4.02
		*COMBOBOX_DEPT*	3.66
		*COMBOBOX_START_TIME*	3.65
		*COMBOBOX_END_TIME*	3.26
	Bigram	*ENVIRONMENT_WB, ENVIRONMENT_MC*	13.20
		*MAIL_MOVE, ENVIRONMENT_WP*	11.95
		*ENVIRONMENT_WB, ENVIRONMENT_WB*	10.87
		*ENVIRONMENT_MC, MAIL_VIEWED_1*	10.34
		*SUBMIT_RESERVATION_FAILURE, HISTORY_MEETINGROOMS*	9.98
	Trigram	*ENVIRONMENT_WB, ENVIRONMENT_WB, ENVIRONMENT_MC*	19.62
		*MAIL_VIEWED_1, MAIL_VIEWED_4, ENVIRONMENT_WB*	18.65
		*ENVIRONMENT_MC, MAIL_VIEWED_1, MAIL_VIEWED_2*	18.29
		*ENVIRONMENT_WB, HISTORY_MEETINGROOMS, ENVIRONMENT_MC*	17.63
		*MAIL_VIEWED_3, MAIL_VIEWED_1, MAIL_VIEWED_4*	15.67
Lower secondary or less	Unigram	*COPY*	30.70
		*MAIL_MOVE*	28.30
		*FOLDER*	22.22
		*PASTE*	20.33
		*FOLDER_VIEWED*	12.20
	Bigram	*MAIL_VIEWED_2, MAIL_MOVE*	31.13
		*MAIL_MOVE, MAIL_VIEWED_3*	28.74
		*FOLDER_VIEWED, MAIL_MOVE*	24.85
		*MAIL_MOVE, MAIL_VIEWED_1*	22.55
		*MAIL_MOVE, MAIL_VIEWED_2*	22.37
	Trigram	*MAIL_MOVE, MAIL_VIEWED_2, MAIL_MOVE*	38.57
		*FOLDER_VIEWED, MAIL_MOVE, MAIL_VIEWED_3*	38.13
		*MAIL_MOVE, MAIL_VIEWED_1, MAIL_MOVE*	30.98
		*MAIL_VIEWED_2, MAIL_MOVE, FOLDER_VIEWED*	29.04
		*MAIL_VIEWED_1, MAIL_MOVE, MAIL_VIEWED_1*	28.07

**Table A14:** Top five features of action sequences selected for tertiary-bachelor degree and lowest education groups.

Group	*N*-gram	Action sequences	Chi-square
Tertiary – bachelor degree	Unigram	*REPLY*	7.10
		*COMBOBOX_ROOM*	6.50
		*COMBOBOX_START_TIME*	5.30
		*SUBMIT_RESERVATION_SUCCESS*	5.14
		*COMBOBOX_DEPT*	4.76
	Bigram	*MAIL_VIEWED_4, MAIL_VIEWED_1*	16.49
		*MAIL_VIEWED_1, MAIL_VIEWED_3*	16.24
		*ENVIRONMENT_WB, ENVIRONMENT_MC*	16.13
		*HISTORY_MEETINGROOMS, ENVIRONMENT_MC*	14.32
		*MAIL_VIEWED_4, MAIL_VIEWED_2*	13.87
	Trigram	*HISTORY_RESERVATION, HISTORY_MEETINGROOMS, HISTORY_VIEWCALENDAR*	20.75
		*ENVIRONMENT_WB, HISTORY_MEETINGROOMS, ENVIRONMENT_MC*	20.25
		*ENVIRONMENT_MC, MAIL_VIEWED_1, MAIL_VIEWED_2*	17.86
		*MAIL_VIEWED_1, MAIL_VIEWED_3, MAIL_VIEWED_4*	16.25
		*MAIL_VIEWED_4, MAIL_VIEWED_1, ENVIRONMENT_WB*	15.25
Lower secondary or less	Unigram	*MAIL_MOVE*	109.87
		*PASTE*	59.04
		*FOLDER_VIEWED*	14.04
		*COPY*	13.82
		*SEARCH*	8.05
	Bigram	*MAIL_VIEWED_1, MAIL_MOVE*	101.94
		*FOLDER_VIEWED, MAIL_MOVE*	98.18
		*MAIL_MOVE, MAIL_VIEWED_2*	90.67
		*MAIL_MOVE, MAIL_VIEWED_3*	90.67
		*MAIL_MOVE, FOLDER_VIEWED*	83.84
	Trigram	*FOLDER_VIEWED, MAIL_MOVE, MAIL_VIEWED_2*	103.42
		*MAIL_MOVE, FOLDER_VIEWED, MAIL_MOVE*	93.20
		*MAIL_MOVE, MAIL_VIEWED_3, FOLDER_VIEWED*	88.33
		*MAIL_VIEWED_3, FOLDER_VIEWED, MAIL_MOVE*	88.33
		*MAIL_VIEWED_3, MAIL_MOVE, FOLDER_VIEWED*	88.13
